# Decentralized digital health ecosystems: a unified architecture for AI-enhanced medical record management

**DOI:** 10.3389/fdgth.2025.1685628

**Published:** 2025-10-27

**Authors:** Harsha Kumar A., Preetham Venkatram C., Saran N., Disha Daniel, Praveen Joe I. R.

**Affiliations:** ^1^ School of Computer Science and Engineering, Vellore Institute of Technology, Chennai, India; ^2^ School of Electronics Engineering, Vellore Institute of Technology, Chennai, India

**Keywords:** blockchain, electronic health records (EHR), artificial intelligence (AI), decentralized autonomous organization (DAO), zero-knowledge proofs (ZKPs), patient-centric healthcare, polygon, interoperability

## Abstract

Traditional Electronic Health Record (EHR) systems suffer from critical vulnerabilities in security, interoperability, and patient data control. This paper introduces PolyMed, a novel decentralized platform designed to address these challenges. PolyMed combines blockchain, Artificial Intelligence (AI), and edge computing into a synergistic architecture. It uses the Polygon blockchain for immutable record-keeping and a Decentralized Autonomous Organization (DAO) for transparent governance. Patient identity is secured through privacy-preserving zero-knowledge proofs (ZKPs) and anchored to non-transferable Soulbound Tokens (SBTs), granting users true sovereignty over their data. The platform also includes a Decentralized Finance (DeFi) module to improve healthcare accessibility. Empirical evaluations on the Polygon Mainnet confirm the system's viability, showing sub-4-second transaction latencies and over 90% cost savings compared to legacy systems. The integrated AI model, leveraging a LightGBM classifier on a rich set of engineered features, achieves an Area Under the Curve (AUC) of **0.8543** and an accuracy of **80.33%** in emergency detection, demonstrating high reliability on a clinically relevant and imbalanced dataset. By aligning with global standards like General Data Protection Regulation (GDPR) and Health Insurance Portability and Accountability Act (HIPAA), PolyMed offers an integrated platform for patient-centric digital health management.

## 1 Introduction

The digital transformation of healthcare, centered on Electronic Health Records (EHRs), promises a future of seamless, data-driven medicine (1). However, the predominantly centralized architecture of current EHR systems presents a fundamental flaw. These systems create single points of failure, making them prime targets for cyberattacks that can cripple hospital operations and compromise patient safety. Furthermore, proprietary data silos prevent interoperability, hindering clinical decision-making and large-scale medical research. In this paradigm, patients lack true ownership of their most sensitive information, creating a critical need for a more secure, interoperable, and patient-centric model.

In response to this increasingly untenable situation, blockchain technology has emerged over the last decade as a powerful and promising architectural alternative. By leveraging a decentralized, cryptographically secure, and immutable ledger, blockchain offers a fundamentally different approach to data management—one that is inherently resilient to single points of failure and resistant to unauthorized tampering. The potential for this technology to revolutionize healthcare was recognized early on by early research projects that provided crucial proofs-of-concept. MedRec, introduced in 2016, was an influential study that demonstrated how smart contracts on the Ethereum blockchain could be used to create a decentralized record management system with a focus on granular, patient-driven permissioning for their medical records (2). Following this, FHIRChain proposed a novel architecture that combined the interoperability benefits of the Fast Healthcare Interoperability Resources (FHIR) standard with the security of a blockchain ledger, aiming to create a system where standardized clinical data could be shared securely and scalably (3). These foundational works were instrumental in establishing the viability of using blockchain for EHRs.

However, these first-generation systems, while innovative, also illuminated a more complex research gap. They primarily focused on the challenges of data storage and access control, leaving several other critical dimensions of a truly patient-centric ecosystem unaddressed. A significant limitation was the handling of digital identity; these systems often relied on raw cryptographic wallet addresses as identifiers, which lack the real-world verifiability required for clinical and legal contexts and do little to prevent impersonation. Furthermore, their architectural models were largely designed for static, episodic health records (like a doctor's visit summary), failing to account for the paradigm shift towards continuous, real-time health monitoring driven by the proliferation of wearable sensors and Internet of Things (IoT) devices. A modern EHR system must be able to securely ingest and analyze these dynamic data streams to enable proactive, preventative care. Finally, these early models did not incorporate frameworks for democratic governance or financial inclusion. They did not answer crucial questions such as: Who decides on the rules for data sharing and system upgrades? And how can technology alleviate the financial barriers that prevent patients from accessing care?

This paper posits that solving these interconnected challenges requires a more sophisticated, synergistic integration of multiple emerging technologies. A truly comprehensive solution for next-generation EHR management must be built on a converged architecture that places the patient at its absolute center. This vision requires a platform that can: (1) cryptographically verify a user's real-world identity in a privacy-preserving manner; (2) intelligently analyze physiological data by engineering descriptive statistical and temporal features to predict and preempt medical emergencies; (3) empower a community of patients and providers to collectively and transparently govern the digital ecosystem; and (4) provide novel financial tools that enhance healthcare accessibility. Addressing this comprehensive research gap is the primary motivation for this research. The paper introduces PolyMed, a holistic, patient-centric EHR management platform architected from the ground up to realize this vision of a secure, intelligent, and empowered digital health ecosystem (the source code for key components is provided (23), as described in the Data and Code Availability Section: 8.1).

The following contributions are made through systematic integration:

• **A Novel Integrated Architecture:** A unified framework is presented that combines the Polygon blockchain, AI-driven analytics, edge computing for health sensor data, a Decentralized Autonomous Organization (DAO) for governance, Zero-Knowledge Proof (ZKP) for identity verification, and Decentralized Finance (DeFi) for microloans. This holistic approach distinguishes PolyMed from prior systems that typically focus on only one or two of these aspects.
• **Privacy-Preserving Verifiable Identity:** The system incorporates Anon-Aadhaar, a ZKP-based solution, to verify user identities without exposing sensitive personal data. This is further secured by binding verified identities to non-transferable Soulbound Tokens (SBTs), ensuring robust, self-sovereign identity management.
• **AI-Powered Clinical Intelligence:** An integrated and validated LightGBM (LGBM) model provides emergency detection from physiological data. By transforming raw time-series data into a comprehensive set of tabular features, the model achieves a high Area Under the Curve (AUC) score on a public clinical dataset, demonstrating the potential for proactive clinical intervention even with highly imbalanced data.
• **Comprehensive Empirical Validation:** A rigorous performance evaluation conducted on the Polygon Mainnet analyzes transaction latency, gas costs, system throughput, and operational resilience. This is complemented by a usability study and a detailed economic analysis, confirming the platform's practical viability and cost-effectiveness.

This paper is organized as follows. Section 2 surveys related work in blockchain-based healthcare. Section 3 details the proposed system architecture and methodology. Section 4 presents the performance analysis and scalability tests. Section 5 discusses the security and compliance frameworks. Section 6 covers the usability and economic implications. Section 7 interprets the findings and discusses limitations, and Section 8 concludes the paper and outlines future work.

## 2 Literature survey

The application of blockchain technology to re-architect EHR systems has been a vibrant and rapidly evolving field of research. The academic literature reflects a clear progression from initial conceptual models to more sophisticated, multi-layered platforms designed to tackle the nuanced challenges of modern healthcare. Literature analysis reveals several key themes that have dominated the discourse, though comprehensive integration remains underexplored: the establishment of foundational architectures for decentralized data sharing; the continuous effort to enhance patient privacy through advanced cryptography; the critical integration of real-time data from the IoT; the persistent drive to solve the blockchain trilemma of scalability, security, and decentralization; and the emerging, yet underexplored, domains of decentralized governance and economic models.

The genesis of this research area was rooted in the fundamental promises of blockchain itself. The core properties of decentralization, which eliminates single points of failure; immutability, which ensures the integrity of the medical record against tampering; and cryptographic transparency, which provides a provably fair and auditable trail of all data interactions, were immediately recognized as powerful antidotes to the vulnerabilities of centralized EHR systems (5). Early researchers grappled with fundamental architectural decisions, such as the trade-offs between permissionless (public) blockchains, which offer maximum transparency, and permissioned (private or consortium) blockchains, which provide greater control over network participants—a critical consideration for a regulated industry like healthcare. The consensus quickly formed around hybrid storage models, recognizing the prohibitive cost and privacy risks of storing voluminous, sensitive health data directly on-chain (1). This led to the dominant architectural pattern where the blockchain is used as a lean, highly secure transaction and access-control layer, while the encrypted data itself resides in off-chain storage.

This foundational work gave rise to the first generation of tangible platforms that served as crucial proofs-of-concept. The MedRec system, emerging from MIT in 2016, provided a landmark demonstration of this hybrid model. It utilized smart contracts on an Ethereum-based ledger to manage a registry of pointers to medical records, which were stored in traditional off-chain databases. Its primary innovation was a sophisticated permissioning system that empowered patients to grant and revoke access to their records for various healthcare providers, creating a patient-mediated audit trail of data access (2). Soon after, FHIRChain addressed another critical dimension: data standardization. While MedRec was data-agnostic, FHIRChain proposed an architecture that intrinsically linked the security of the blockchain to the interoperability of the Fast Healthcare Interoperability Resources (FHIR) standard (3). This was a conceptual advancement, as it envisioned a future where standardized clinical data could be shared not just securely, but also meaningfully, between disparate systems. While these pioneering systems were instrumental, their focus remained primarily on the access control of static, episodic health records, and they generally relied on pseudonymous wallet addresses for identity, which lack the real-world, verifiable credentials required for clinical practice.

As the field matured, the research focus intensified on strengthening the privacy and security guarantees of these systems. The inherent transparency of many blockchains presented a privacy paradox, leading researchers to explore advanced cryptographic solutions. One prominent avenue of exploration has been fully homomorphic encryption, a powerful technique that allows for mathematical computations to be performed on encrypted data without ever needing to decrypt it. This holds immense potential for privacy-preserving analytics, where a healthcare provider could, for instance, outsource complex data analysis to a third-party cloud service without exposing the underlying patient information (6). However, the significant computational overhead associated with current homomorphic encryption schemes has largely confined them to theoretical or niche applications, limiting their use in high-throughput, real-time clinical environments. This performance bottleneck has fueled a growing interest in more efficient privacy-enhancing technologies, particularly Zero-Knowledge Proofs (ZKPs). ZKPs offer a notable capability: the ability to prove the validity of a statement without revealing the underlying data that supports the statement (7–9). This is perfectly suited for healthcare use cases, such as a patient proving they have a valid prescription to a pharmacy without revealing their name or diagnosis. Recent research has begun to explore how ZKPs can enable large-scale, privacy-preserving data analytics, a critical requirement for advancing public health research without compromising individual patient confidentiality (10).

Parallel to the advancements in cryptography, another major research thrust has been the integration of real-time data from the burgeoning Internet of Medical Things (IoMT). The proliferation of wearable sensors, smart medical devices, and remote patient monitoring tools has fundamentally changed the nature of health data from static and episodic to continuous and dynamic. This has created an urgent need for architectures capable of securely ingesting, storing, and analyzing these high-velocity data streams. The synergy between IoMT and blockchain is a powerful one; blockchain can provide an immutable, auditable record of data originating from a distributed network of devices, ensuring its provenance and integrity (11). The necessity of pairing these trusted data streams with Machine Learning (ML) and Artificial Intelligence (AI) for proactive care—such as early disease detection, chronic condition management, and real-time emergency alerts—has become a central theme in many recent studies (12, 13). However, the sheer volume of IoMT data makes direct on-chain storage infeasible. To solve this, edge computing has emerged as a critical architectural component. By deploying computational resources at the network edge, closer to the patient, data can be pre-processed, filtered, and analyzed locally. This approach reduces latency for time-sensitive alerts, minimizes the data load on the core network, and enhances security by ensuring that only relevant and validated information is transmitted to the blockchain layer (14).

The inherent challenge of achieving scalability without sacrificing decentralization or security—often referred to as the “blockchain trilemma”—has been a constant driver of innovation in this space. The high transaction fees and low throughput of early blockchains like Ethereum Classic made them unsuitable for large-scale healthcare applications. In response, the field has widely adopted solutions built on more scalable platforms and Layer-2 technologies. High-throughput blockchains and sidechains, such as Polygon, have become popular choices due to their low transaction costs and compatibility with the Ethereum Virtual Machine (EVM), making it possible to build complex decentralized applications that are economically viable (15). The hybrid, “thin blockchain” model, where the ledger is used for high-value transactions like identity verification and access control while bulk data is stored off-chain, is now a standard design pattern (16, 17). Comprehensive frameworks have been proposed to bundle these components into integrated platforms (4, 18), and multi-chain solutions are being explored to further enhance trust and transparency in complex, multi-stakeholder healthcare environments (19).

Despite this impressive and multifaceted body of research, a comprehensive review of the literature reveals two critical, yet largely unaddressed, pillars of a truly patient-centric ecosystem: decentralized governance and integrated economic models. The vast majority of prior work has focused on solving the technical challenges of data management, while implicitly assuming a centralized or consortium-based model for governance. Critical questions—such as who sets the rules for data access, how the protocol is upgraded, and how disputes are resolved—were often left unanswered. The emergence of Decentralized Autonomous Organizations (DAOs) offers a powerful solution, enabling communities of stakeholders to collectively and transparently govern a digital platform (5). Similarly, while the technical aspects of healthcare are well-studied, the economic barriers to accessing care are often overlooked in these systems. The rise of Decentralized Finance (DeFi) presents a new frontier of programmable, transparent financial tools that could be integrated into healthcare platforms to provide novel solutions like microloans for medical expenses, automated insurance claims processing, and staking mechanisms to fund community health initiatives (20). The integration of these social, governmental, and economic layers into a technically robust EHR framework remains an active research area.

In summary, while substantial progress has been made in individual domains, a comprehensive system that integrates privacy-preserving verifiable identity, real-time AI-driven clinical intelligence, genuine patient-led governance, and integrated financial inclusion into a single, cohesive, and empirically validated framework has remained an open and significant research gap. Table 1 provides a detailed comparative analysis of key existing systems against the proposed architecture, PolyMed, to visually and conceptually summarize this research gap and underscore the novelty of the integrated approach.

**Table 1:** Comparative analysis of Key blockchain-based EHR systems.

Feature	MedRec (2)	FHIRChain (3)	BC IoMT U6 HCS (4)	PolyMed (Proposed)
Data storage	Off-chain with on-chain metadata/hashes.	Off-chain with on-chain FHIR resource pointers.	Cloud storage with blockchain for access control.	IPFS storage with on-chain hashes and edge computing pre-processing.
Identity management	Ethereum addresses. Relies on external identity verification.	Not explicitly detailed; assumes pre-verified identities.	Traditional authentication methods.	ZKP-based identity verification (Anon-Aadhaar) and non-transferable Soulbound Tokens (SBTs).
IoT integration	Not a primary focus. Data is primarily static EHR entries.	Not a primary focus. Designed for FHIR resources, not real-time streams.	Yes, focuses on IoMT data integrity and authentication.	Deep integration with real-time health data streams via a dedicated edge layer for filtering and anomaly detection.
Governance	Centralized system administrators or consortium-based.	Centralized or consortium-based.	Centralized control over the framework.	Fully decentralized governance via a token-based Decentralized Autonomous Organization (DAO).
Financial inclusion	Not addressed.	Not addressed.	Not addressed.	Integrated DeFi module for on-chain microloans and staking for medical expenses.
AI integration	Not included.	Not included.	Not included.	Validated AI model for real-time emergency detection and an architecture for AI-driven scheduling and patient assistance.

## 3 System architecture and methodology

The PolyMed platform is architected as a multi-layered, decentralized system meticulously designed to address the foundational challenges of security, interoperability, privacy, and patient empowerment in modern Electronic Health Record (EHR) management. The architecture is not a monolithic application but rather a synergistic composition of several core technological pillars, each selected for its specific capacity to solve a distinct problem within the healthcare data ecosystem. The design philosophy is rooted in the principles of **privacy-by-design**, **patient-centricity**, and **zero-trust**, where control is cryptographically guaranteed and distributed among stakeholders rather than being concentrated in a single administrative entity. This section provides an exhaustive exploration of this architecture, beginning with its high-level design philosophy, delving into the technical intricacies of its core technological pillars, detailing the specific components and their end-to-end workflows, and finally, dissecting the advanced modules that provide PolyMed with its intelligent, autonomous, and financially inclusive capabilities.

### 3.1 Architectural philosophy and high-level design

PolyMed's design is based on a fundamental goal: giving patients full control over their own health data. This principle of **patient data sovereignty** is the platform's core philosophical commitment. Unlike traditional systems where providers control the data, PolyMed uses strong cryptography to guarantee that only the patient can own and share their records. To ensure this guarantee is secure, the system relies on strong encryption. The strength of this protection is formally measured by entropy $\left(H_{\mathrm{key}}\right)$, where a higher value means it is exponentially harder for an attacker to guess the key. The entropy is directly proportional to the key's length in bits $\left(L_{\mathrm{bits}}\right)$:

(1) $$H_{\mathrm{key}}=L_{\mathrm{bits}}$$

By employing 256-bit keys, PolyMed establishes a foundation that is secure against all known brute-force attacks with current and foreseeable computing technology. Every patient's record is linked to their private key, and no access or transaction can occur without their explicit, digitally signed consent. This paradigm shift aims to rebalance the power dynamic in healthcare data, fostering a new level of trust and transparency between patients and the healthcare ecosystem.

To achieve this without compromising scalability or privacy, a **thin blockchain** design philosophy was adopted. This architectural pattern dictates a strategic separation of concerns between on-chain and off-chain environments. The blockchain layer, while being the system's core trust anchor, is used sparingly and efficiently. It is reserved exclusively for operations that require absolute immutability, transparency, and decentralized validation: identity verification, access control permissions, critical metadata (such as record hashes), and the execution of governance and financial logic via smart contracts. The voluminous and highly sensitive EHR data itself—such as clinical notes, lab results, and high-resolution medical imagery—is never stored directly on the blockchain. This hybrid model provides the best of both worlds: the strong security and auditability of a blockchain for trust-sensitive operations, and the scalability, cost-effectiveness, and privacy of off-chain storage for bulk data. This approach directly addresses the prohibitive costs and inherent privacy risks associated with storing large datasets on a public or semi-public ledger.

The architecture also draws inspiration from established software engineering principles, adapted for a decentralized context. The design of the smart contracts and off-chain services adheres to the **SOLID principles**. For example, each smart contract is designed with a **Single Responsibility**, such as the “AuthSC” focusing exclusively on identity and the “DeFiLoanSC” on financial logic. This modularity enhances security and simplifies auditing. The contracts are designed to be extensible via proxy patterns but immutable in their core logic, embodying the **Open/Closed Principle**. Furthermore, interactions between contracts and between on-chain and off-chain components occur through well-defined, minimal interfaces, reflecting the **Interface Segregation Principle** and reducing the attack surface of the system.

The overall system can be conceptualized in several distinct, interacting layers. The **Presentation Layer** consists of the user-facing web application, providing an intuitive interface for patients, clinicians, and administrators to interact with the system's functionalities. The **Logic Layer** is composed of the suite of smart contracts deployed on the Polygon blockchain, which encode the core business rules, governance mechanisms, and financial protocols of the ecosystem. The **Data Layer** is a hybrid construct, comprising the Polygon blockchain for immutable metadata and pointers, and the InterPlanetary File System (IPFS) for the distributed storage of the actual encrypted EHR data. Finally, the **Intelligence Layer** consists of the off-chain Artificial Intelligence (AI) and machine learning models that provide clinical decision support. For this study, time-series data is first converted into a rich set of tabular features (e.g., statistical summaries, trends). A powerful gradient boosting model then analyzes this feature set to make predictions. Because blockchains cannot natively access external data, this layer interacts with on-chain contracts through a secure oracle system. This addresses the fundamental “oracle problem” by providing a trusted bridge to bring off-chain computational results, such as an AI-driven emergency assessment, into the deterministic on-chain environment. These layers are designed with principles of loose coupling and high cohesion, allowing for modular development, independent scalability, and easier maintenance.

**Figure 1:**
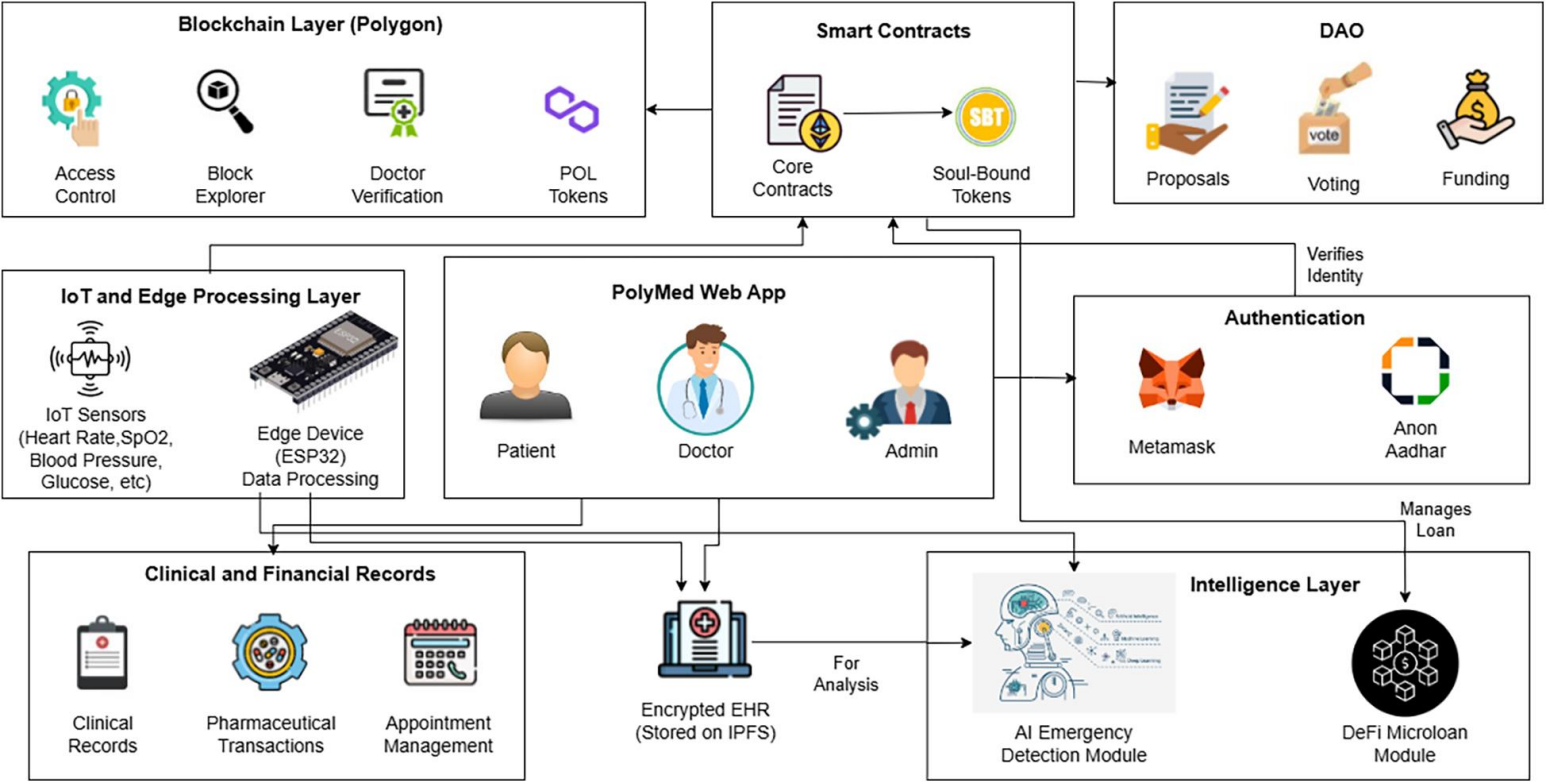
The overall system architecture of PolyMed. This diagram provides a holistic view of the platform, illustrating the interaction between end-users (patients, doctors), the web application frontend, the edge computing layer for pre-processing IoT data, the Polygon blockchain which hosts the core smart contracts and DAO, and the decentralized storage layer (IPFS) for encrypted EHR data. Each component is designed to work synergistically to create a secure, patient-centric health ecosystem.

**Figure 2:**
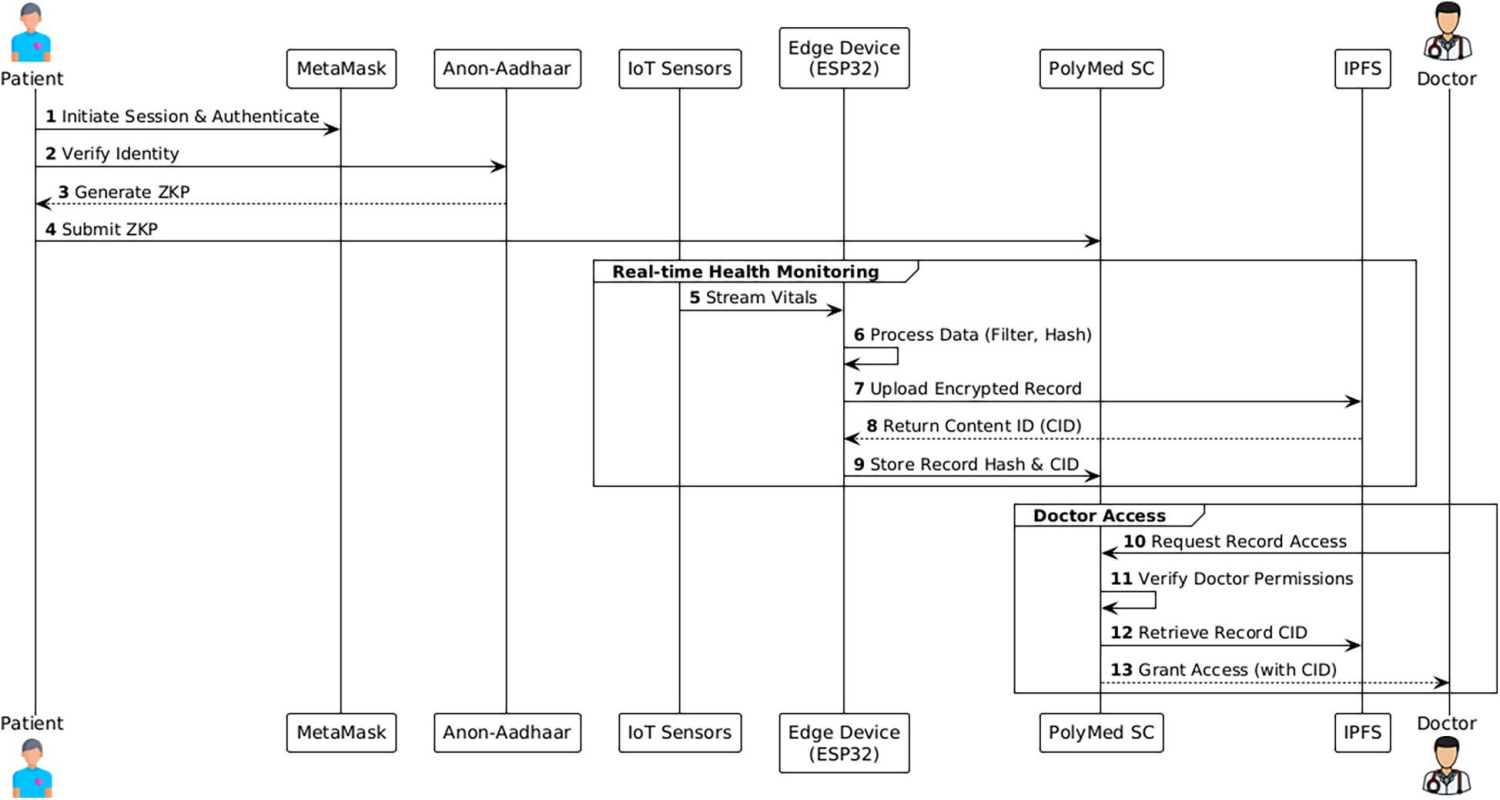
System workflow sequence diagram. This diagram illustrates a typical user interaction flow, detailing the sequence of operations from patient authentication to a doctor accessing a medical record. (1) The patient initiates a session, authenticating via MetaMask and verifying their identity with an Anon-Aadhaar ZKP. (2) IoT sensors stream vitals to the edge device. (3) The edge device processes the data and sends a validated hash to the PolyMed smart contracts on Polygon. (4) Later, a doctor authenticates and requests access. (5) The smart contract verifies the doctor's permissions and retrieves the data hash and IPFS link for the encrypted record, granting access.

The core components of the Logic Layer are a suite of specialized smart contracts, each with a distinct function, as summarized in Table 2. The high-level interaction between these components is illustrated in the overall system architecture diagram (Figure 1). A more dynamic view of these interactions is provided in the workflow sequence diagram (Figure 2), which traces a typical user journey from the initial, privacy-preserving authentication process through to the secure retrieval of a medical record by an authorized clinician, showcasing the end-to-end flow of control and data within the PolyMed ecosystem.

**Table 2:** Key smart contracts and their functions in the PolyMed ecosystem.

Smart contract	Primary functions	Description
AuthSC	User authentication, ZKP verification, SBT minting, emergency access control	Manages user identities and access permissions, serving as the gatekeeper for the entire system.
EHRDataSC	EHR storage (hashes/CIDs), record retrieval, access logging	Core contract for managing the pointers and metadata associated with patient health records stored on IPFS.
AppointmentSC	Appointment scheduling, doctor availability management	Automates the booking and management of medical appointments between patients and verified clinicians.
PrescriptionSC	Prescription issuance, verification, fulfillment tracking	Handles the lifecycle of digital prescriptions, ensuring they are issued by verified doctors and fulfilled securely.
DAOGovernanceSC	Proposal submission, voting, treasury management, dispute resolution	Facilitates decentralized governance, including the management of system parameters and the economic policies of the DeFi module.
DeFiLoanSC	Microloan requests, disbursement, repayment, staking	Manages financial services for patients, allowing them to access undercollateralized loans for medical expenses funded by community liquidity providers.
IoTDataSC	IoT data ingestion (validated/hashed), alert flagging	Processes and secures real-time data from authenticated IoMT devices, creating an immutable on-chain log of vital measurements.

### 3.2 Polygon blockchain: a deep dive into the trust layer

While other scalable blockchain solutions like optimistic rollups and alternative Layer-1s exist, the decision to build PolyMed on Polygon was driven by a confluence of four key advantages:

1. **EVM Compatibility and a Mature Ecosystem:** Polygon is fully compatible with the Ethereum Virtual Machine (EVM). This is arguably its most significant advantage, as it means smart contracts can be written in Solidity, the most widely used and well-audited smart contract language. More importantly, it grants access to the entire, unparalleled ecosystem of Ethereum development tools (Truffle, Hardhat), security libraries (OpenZeppelin), and infrastructure providers (Infura, Alchemy). This drastically reduces development time, lowers the risk of introducing novel vulnerabilities, and ensures long-term access to a massive global pool of developer talent.
2. **High Throughput and Low Transaction Costs:** Compared to Ethereum's 15–30 Transactions Per Second (TPS), Polygon can theoretically handle up to 7,000 TPS, with practical throughput being in the hundreds. This is more than sufficient to handle the transactional load of a large network of hospitals and patients. Furthermore, transaction costs on Polygon are typically orders of magnitude lower than on Ethereum, often costing fractions of a cent. This economic viability is non-negotiable for a healthcare system, as it ensures that core functionalities like updating a health record or granting access remain affordable and accessible to all users.
3. **Robust Security and Decentralization:** The consensus and security are managed by the **Heimdall layer**. This layer consists of a set of validators who participate in a Proof-of-Stake (PoS) consensus mechanism. To do so, they “stake” their own MATIC tokens, which acts like a security deposit; this economic incentive ensures they validate transactions honestly to protect the network. These validators periodically bundle up blocks and commit a cryptographic “checkpoint” to the Ethereum mainnet. This checkpointing mechanism is crucial, as it allows the Polygon chain to periodically anchor its state to the strong security of Ethereum, making it extremely difficult to reverse or tamper with transactions.
4. **Interoperability and Future-Proofing:** As a key part of the Ethereum ecosystem, Polygon is at the forefront of research into future scaling solutions, including ZK-rollups. By building on Polygon, PolyMed is well-positioned to take advantage of these future technologies, ensuring the long-term scalability and relevance of the platform.

### 3.3 Self-Sovereign identity: a paradigm shift with ZKPs and SBTs

A cornerstone of the PolyMed architecture is its novel approach to digital identity, which moves away from traditional, centralized models towards a paradigm of **Self-Sovereign Identity (SSI)**. SSI is a model where individuals have sole control over their own digital identities, without depending on any intermediary or central authority. It is built on the principles of user control, consent, data minimization, and portability. In the context of healthcare, SSI is transformative because it allows a patient to own, manage, and share their verifiable health credentials in a secure and granular way. PolyMed implements SSI by integrating two powerful cryptographic and tokenomic primitives: Zero-Knowledge Proofs (ZKPs) and Soulbound Tokens (SBTs).

Furthermore, the structure of the identity objects is designed with an eye toward compatibility with the emerging **W3C Verifiable Credentials (VC)** data model. While SBTs provide a simple and robust on-chain representation, the off-chain data that is cryptographically proven can be structured as a VC, containing claims, metadata, and a digital signature from an issuer (e.g., a government or a medical board). This ensures that while the system is self-contained, it is also future-proof and capable of interoperating with a broader, standards-based digital identity ecosystem.

**Zero-Knowledge Proofs** represent a breakthrough in cryptography and are the engine of PolyMed's privacy-preserving verification system. A ZKP allows a “prover” to convince a “verifier” that they know a secret or that a statement is true, without revealing the secret or any other information whatsoever. This is achieved using a type of non-interactive ZKP known as a **zk-SNARK** (Zero-Knowledge Succinct Non-Interactive Argument of Knowledge), which produces proofs that are very small and fast to verify, making them ideal for on-chain applications. In conceptual terms, this process is analogous to proving knowledge of a password by providing a valid cryptographic hash of it, without revealing the password itself. The computational cost of generating these proofs, $C_{\mathrm{ZKP}\_\mathrm{gen}}$, is a critical factor and can be approximated as being quasi-linear in the number of constraints in the ZKP circuit:

(2) $$C_{\mathrm{ZKP}\_\mathrm{gen}}=O(N_{\mathrm{constraints}}\cdot \log N_{\mathrm{constraints}})$$

where $N_{\mathrm{constraints}}$ is the number of Rank-1 Constraint System (R1CS) constraints in the circuit, a detail discussed in foundational works such as (7). For this implementation, the **Anon-Aadhaar SDK** is integrated. The Aadhaar system in India provides a unique digital identity to over a billion people. Anon-Aadhaar leverages this by allowing a user to generate a zk-SNARK locally on their device. The user scans the QR code on their Aadhaar card, which contains their digitally signed demographic data. A ZKP circuit then processes this data to generate a proof that attests to certain facts (e.g., “I am a unique person in the database,” or “I am over the age of 18”) without revealing the underlying name, date of birth, or Aadhaar number. This proof is then submitted to a verifier smart contract on the Polygon blockchain. The smart contract can verify the proof's validity in a gas-efficient manner, confirming the user's identity without ever having access to their Personal Identifiable Information (PII). This verification is a boolean function, formally represented as:

(3) VerifyZK(Proof,PublicInput)=true/false

Once a user's identity is verified via a ZKP, this verification needs to be represented on-chain in a persistent and non-speculative manner. For this, **Soulbound Tokens (SBTs)** are used. An SBT is a type of non-fungible token (NFT) that is designed to be non-transferable. Once an SBT is minted to a specific wallet address (a “soul”), it cannot be sold, gifted, or otherwise transferred to another wallet. This non-transferability makes them perfect for representing personal credentials, achievements, and affiliations that define an identity. In PolyMed, upon successful ZKP verification, the “AuthSC” smart contract mints a role-specific SBT to the user's wallet. These SBTs function as a persistent, on-chain passport, allowing smart contracts throughout the ecosystem to instantly and efficiently check a user's role and permissions simply by querying the presence of a specific SBT in their wallet. This entire authentication flow is specified in Algorithm 1.

A similar credentialing process applies to healthcare providers. A clinician would submit their verifiable credentials (e.g., medical license) to a DAO-governed verification contract. Upon successful off-chain validation by a designated committee, a “Doctor” role SBT would be minted to their wallet, granting them the necessary permissions within the system. This ensures that only verified professionals can access patient data.

Algorithm 1 **Auth Layer Workflow: MetaMask & Anon-Aadhaar Integration**

1: **Init:** *u* (end-user), *w* (wallet addr), qr (Aadhaar-QR), *z* (ZK-proof), sc (Auth SC)

2: **function** connectWallet()

3:  *w* ← Metamask.connect()

4:  *n* ← randNonce()

5:  *σ* ← Metamask.sign(*n*)

6:  **if** ecrecover (n,σ)==w **then**

7:   session.valid ← **true**

8:  **else**

9:   **return fail**

10:   **end if**

11: **end function**

12: **function** genProof(*qr*)

13:  *z* ← AnonAadhaar.generateZK(*qr*)

14:  **return** z

15: **end function**

16: **function** submitProof(z)

17:  **if** sc.verifyZK(w,z) == **true then**

18:   sc.mintSBT(w) ▹ Soul-bind the identity

19:   userTable [w] ← verified

20:  **else**

21:   **return fail**

22: **end if**

23: **end function**

/* Emergency-only shortcut for clinicians */

24: **function** chkEmergency(w)

25:  **if** userTable [w] == verified **then**

26:   **return grant_access**

27:  **else**

28:   **return deny**

29:  **end if**

30: **end function**

### 3.4 The IoMT data pipeline: from sensor to ledger

The secure and efficient management of real-time data from the Internet of Medical Things (IoMT) is a core functional requirement of the PolyMed platform. While the core focus of the empirical validation was the on-chain and AI software components, a representative hardware prototype was constructed to ensure the data pipeline was designed and tested for a realistic, real-world use case. The IoT sensor node was architected around the ESP32 microcontroller, selected for its integrated Wi-Fi and Bluetooth Low Energy (BLE) capabilities. This node was designed to interface with a suite of common medical-grade sensors to capture a wide range of vital signs, including an ECG/EEG monitor for cardiac and neural activity, a glucometer for blood glucose, a pulse oximeter for $\mathrm{Sp}O_{2}$ and heart rate, a clinical-grade temperature sensor, and a digital blood pressure monitor. This prototype was subsequently used to generate realistic and diverse time-series data that served as the input for testing the edge processing logic and for validating the on-chain transaction throughput of the system. The cryptographic chain of custody starts at the point of capture: each data packet transmitted from the sensor can be digitally signed with a private key embedded in the device's secure hardware module. This signature ensures the data's origin and integrity.

Once onboarded, the device begins capturing physiological data, with the **ESP32 microcontroller itself acting as the local edge computing device**. This **Edge Processing** is algorithmically sophisticated and serves multiple purposes:

1. **Noise Filtering and Signal Enhancement:** Raw sensor data is often noisy. The quality of the signal can be formally measured by the Signal-to-Noise Ratio (SNR):
  (4) $$\mathrm{SNR}\;(V)=10\log_{10}\left(\frac{P_{\mathrm{signal}}}{P_{\mathrm{noise}}}\right)$$
  where $P_{\mathrm{signal}}$ is the power of the physiological signal and $P_{\mathrm{noise}}$ is the power of the noise. The edge layer applies digital signal processing algorithms, such as a 4th-order Butterworth filter, to improve this ratio.
2. **Data Aggregation and Summarization:** To reduce the sheer volume of data, the edge device performs aggregation.
3. **Real-Time Anomaly Detection:** The edge device runs lightweight anomaly detection algorithms, such as an Exponentially Weighted Moving Average (EWMA) model, which adapts to a patient's changing baseline.
4. **Data Serialization and Encryption:** Before any data leaves the edge device, it is serialized into a compact binary format, end-to-end encrypted using Advanced Encryption Standard (AES)-256, and then signed by the edge device's private key.

**Mitigation Framework for Connectivity and Power Constraints.** To address the real-world challenges of intermittent connectivity and power limitations inherent in wearable devices, a systematic mitigation framework is integrated into the edge layer. This framework employs a three-pronged strategy: (1) **Edge Caching and Store-and-Forward:** The ESP32 device utilizes its flash memory as a temporary buffer to cache encrypted data packets during periods of network unavailability. Once connectivity is restored, a store-and-forward mechanism transmits the buffered data in chronological order, ensuring no data is lost. (2) **Offline-First Synchronization:** The device operates in an “offline-first” mode, where all critical data processing and anomaly detection occur locally without requiring a constant network connection. Data synchronization is treated as a background task that executes opportunistically. (3) **Adaptive Power Management:** The device implements an energy efficiency protocol that adjusts the data transmission frequency based on the device's battery level and the clinical stability of the patient's vitals. For instance, transmission frequency is reduced during periods of normal readings and low battery, conserving power for critical events.

Only after this rigorous pre-processing is the data ready for the final stage. For this prototype, standard clinical thresholds were used for anomaly detection; however, in a production system, these thresholds would be configurable on a per-patient basis as determined by a clinician. The edge device uploads the encrypted data blob to the **InterPlanetary File System (IPFS)**. The edge device then initiates a transaction on the Polygon network, calling a function on the “IoTDataSC” smart contract and passing it the resulting Content Identifier (CID). The transaction itself is signed by the patient's private key, completing the cryptographic chain of custody. This entire monitoring and data validation workflow is formalized in Algorithm 2.

Algorithm 2 **IoT-Blockchain-Based Health Monitoring and Alerting Workflow**

1: **Init:** S←[HR, SpO_2_, BP, Glu]

2:    T←{HR:(40,160), SpO_2_:(85,100), BP:[90/60,180/120],      Glu:(70,200)}

3:    PID←Patient ID, DID←Device ID

4:    SC←SmartContract

5: **Constants:** DATA_INTERVAL = 10s

6: **function** Initialize (PID)

7:  Register DID to PID on blockchain

8:  Bind sensors *S* to DID, authorize with SC

9: **end function**

10: **function** Monitor(DID)

11:  **while** true **do**

12:    D←ReadVitals()

13:   **if** Validate(D) **then**

14:     E←Encrypt(D,PID)

15:    SC.StoreData(PID,E)

16:    Log“OK”, time(“OK”, time)

17:   **else**

18:    TriggerAlert(PID,D)

19:    Log“Anomaly”, time(“Anomaly”, time)

20:   **end if**

21:   SleepDATA_INTERVAL(DATA_INTERVAL)

22:  **end while**

23: **end function**

24: **function** Validate(D)

25:  **return** T.HR[0]≤D.HR≤T.HR[1] **and**

   $T.SpO_{2}[0]\leq D.SpO_{2}\leq T.SpO_{2}[1]$ **and**

   T.BP[0]≤D.BP≤T.BP[1] **and**

   T.Glu[0]≤D.Glu≤T.Glu[1]

26: **end function**

27: **function** TriggerAlert(PID,D)

28:  Emit blockchain event: EMR(PID,D)

29:  Notify caregiver

30:  SC.Flag(PID,D)

31: **end function**

### 3.5 Core EHR and clinical workflows

The management of clinical data and workflows is orchestrated by a set of interconnected smart contracts that handle everything from record creation to prescription fulfillment. The core workflow for creating, storing, and accessing EHRs is governed by smart contracts to ensure patient consent and data integrity. Patient data is encrypted client-side using a strong symmetric encryption scheme like AES-256, formally represented as:

(5) $$E=\mathrm{Encrypt}\;(\mathrm{Data},K_{\mathrm{sym}})$$

where $K_{\mathrm{sym}}$ is a symmetric key exclusively managed by the patient, often derived from their wallet's signature. The hash of this encrypted data, H(E), along with the IPFS CID, is then stored on-chain to provide a tamper-proof seal of integrity:

(6) OnChainData={PatientID,Timestamp,H(E),IPFS_CID}

This ensures that while the data remains private off-chain, its integrity and existence can be publicly and irrefutably verified against the on-chain record. The general logic for these clinical interactions is specified in Algorithm 3.

Algorithm 3 **Secure EHR Management Workflow Logic**

1: **Init:** p,d,iot,b,ph,pid,did

2: *data* ← [“heart”, “oxygen”, “bp”, “glucose”]

3: **function** register*p*(*p*)

4:   **if** p∉b **then**

5:    Auth, Add(*pid*,*b*)

6:   **else**

7:    Auth

8:   **end if**

9: **end function**

10: **function** collect()

11:   **while** true **do**

12:     d←iot[pid].capture()

13:    **if** valid(*d*) **then**

14:     Send(*d* → *b*)

15:    **else**

16:     Discard

17:    **end if**

18:   **end while**

19: **end function**

20: **function** appoint()

21:   **if** Req(*did*) **then**

22:    Get(*rec*), Analyze, Store

23:   **else**

24:    Notify

25:   **end if**

26: **end function**

27: **function** purchase()

28:   **if** hasRx && verify(*rx*) **then**

29:    Notify(*ph*), Log(*rx* → *b*)

30:   **else**

31:    Notify

32:   **end if**

33: **end function**

34: **function** emergency()

35:   **while** isEmerg(*d*) **do**

36:    Alert, Act, Update(*b*)

37:   **end while**

38: **end function**

39: **function** isEmerg*d* (*d*)

40:   **return** abnormal vitals

41: **end function**

### 3.6 Advanced system modules and governance

Beyond the core infrastructure for identity and data management, PolyMed incorporates several advanced modules that provide its unique intelligent, autonomous, and financially inclusive capabilities. These modules are designed to be interoperable and composable, building upon the foundational layers of identity and data.

The integration of Artificial Intelligence is central to PolyMed's vision of proactive healthcare. The primary component of the intelligence engine is the AI model for emergency detection. This model is a **LightGBM (LGBM)** classifier, a highly efficient gradient boosting framework adept at handling tabular data. The architectural workflow involves an initial feature engineering step where raw time-series data is converted into a structured feature set. This tabular data is then fed into the LGBM model, which is deployed on a secure off-chain server. For the purposes of this prototype, this server functions as a centralized, trusted oracle. However, a production-grade deployment would require a decentralized approach to eliminate this single point of trust. This could be achieved by leveraging a decentralized oracle network like **Chainlink**, which could fetch the AI model's output from multiple independent nodes. The primary challenges in such a decentralized system include ensuring **verifiable computation** (cryptographically proving that the correct model was executed on the correct data), designing robust **economic incentives** for the AI node runners, and preserving the privacy of the model's intellectual property.

This server runs a listener service that continuously monitors the Polygon blockchain for events emitted by the “IoTDataSC” smart contract. If the model classifies the situation as “Critical,” it can trigger a high-priority alert back to the blockchain. The end-to-end workflow relies on a suite of specialized smart contracts and oracles. These include core contracts for authentication $\left(C_{\mathrm{Auth}}\right)$ and data management $\left(C_{\mathrm{Data}}\right)$, alongside specific contracts for payments $\left(C_{\mathrm{Pay}}\right)$, cross-chain interactions $\left(C_{X}\right)$, and immutable logging $\left(C_{\mathrm{Log}}\right)$. The system also leverages oracles to securely bring real-world information on-chain, including a health oracle for emergencies $\left(O_{H}\right)$, a reputation oracle $\left(O_{\mathrm{Rep}}\right)$, and a market oracle for pricing $\left(O_{\mathrm{Mkt}}\right)$. This alert can, in turn, call specialized on-chain contracts. These contracts can grant emergency services temporary data access for a duration managed by DAO governance (e.g., EMERGENCY_ACCESS_DURATION) and, crucially, can also interact with the DeFi module. For instance, a verified on-chain emergency event could automatically notify the patient of their pre-assessed eligibility for a microloan, streamlining their access to funds for urgent treatment and demonstrating a seamless link between clinical events and financial support. The full end-to-end flow, integrating authentication, data access, and AI-triggered emergency responses, is detailed in Algorithm 4.

Algorithm 4 **Cross-Chain Healthcare Flow Integrating ZKP Authentication & AI-Driven Operations**

1: **Input:** ID,R,zkP,op,Sig,AI

2: **Contracts:** $C_{Auth},C_{Data},C_{Pay},C_{X},C_{Log}$

3: **Oracles:** $O_{H},O_{Rep},O_{Mkt}$

4: **Constants:** EMERGENCY_ACCESS_DURATION = 1h

5: **function** AuthZK(ID,zkP,R)

6:    $ok\leftarrow C_{Auth}.checkZKP(ID,zkP,R)$

7:   **require** ok **else revert**

8:   mintSBT(ID,R)

9: **end function**

10: **function** RunOp(ID,op)

11:   **if** op=‘‘appt" **then**

12:     $root\leftarrow C_{Data}.getRoot("doc")$

13:    **require** verifyMP(root) **else revert**

14:     t←AI.pickTime()

15:     $slot\leftarrow C_{Data}.book(ID,t)$

16:    emit“Booked”, ID,slot (“Booked”, ID,slot)

17:   **else if** op=‘‘buy" **then**

18:     $rx\leftarrow C_{Data}.getRx(ID)$

19:    **require** decryptRx(rx,zkP) **else revert**

20:     $p\leftarrow O_{Mkt}.price(rx.drug)$

21:     $C_{Pay}.pay(ID,"Pharma",p)$

22:     $C_{X}.logRx(ID,rx.hash())$

23:   **else if** op=‘‘emg" **then**

24:     $v\leftarrow C_{Data}.vitals(ID)$

25:     crit←AI.isCritical(v)

26:    **require** crit **else revert**

27: $C_{Auth}$.grant(“EMT”, ID, EMERGENCY_ACCESS_DURATION)

28:     $O_{H}.callAmbulance(ID)$

29:   **end if**

30:    $C_{Log}.log(op,ID,O_{Rep}.score(ID))$

31: **end function**

Looking toward the future, this feature-engineering-first architecture is designed to support more advanced, privacy-preserving machine learning paradigms. A significant avenue for future work is the implementation of **Federated Learning**. In a federated model, the feature engineering pipeline would be executed locally within each participating hospital or clinic. A global model could then be trained on these anonymized, aggregated feature sets without the raw patient data ever leaving the secure confines of the local institution. This approach would allow the PolyMed AI engine to learn from a diverse, multi-institutional dataset—improving its accuracy and reducing bias—while maintaining the highest standards of patient privacy.

To achieve true decentralization, a system needs a mechanism for distributed decision-making and evolution. PolyMed achieves this through a **Decentralized Autonomous Organization (DAO)**. The PolyMed DAO is a set of smart contracts that allow the community of stakeholders to govern the platform collectively. The entire lifecycle of a proposal is managed on-chain, ensuring transparency and censorship resistance. The DAO operates on a principle of **token-weighted voting**, where the influence of a vote is proportional to the number of tokens the voter has staked in the governance contract. The voting power, $V_{p}$, of a participant is directly proportional to their staked tokens, $T_{p}$:

(7) $$V_{p}=\alpha \cdot T_{p}$$

where α is a weighting factor. A proposal is approved if the cumulative voting weight of affirmative votes surpasses a predefined quorum threshold, Q:

(8) $$\sum_{\;p\in \mathrm{YesVotes}}V_{p}\geq Q$$

The DAO acts as the steward of the ecosystem's economic health, with its primary tool being the control over a central **Treasury**. The treasury is a smart contract that holds a pool of community-owned funds. These funds can be sourced from various mechanisms, such as a small percentage of the interest generated by the DeFi module, fees for specific enterprise-level services, or initial token allocations. The DAO has sole control over the treasury, and funds can only be spent if a proposal to do so is passed by the token holders. To mitigate the risk of governance attacks or plutocracy, future iterations of the DAO could explore more advanced voting mechanisms like **quadratic voting**. The core logic of governance and identity management is specified in Algorithm 5.

**DAO Onboarding and Education Framework.** To address the “DAO literacy gap” identified in usability studies, a concrete onboarding framework is proposed. This framework is designed to educate and empower all stakeholders, regardless of their technical background. It consists of three main components:

• **Interactive Tutorials:** A series of guided, in-app tutorials will walk new users through the core concepts of the DAO, including how to view proposals, the mechanics of token-weighted voting, and how to submit a proposal.
• **User-Friendly Guides and Documentation:** A dedicated section of the platform will host comprehensive, non-technical documentation with clear examples and FAQs explaining the governance process, the role of the treasury, and the impact of key proposals.
• **Incentivized Participation Program:** To encourage active engagement, a rewards program will be implemented. Users will earn small amounts of governance tokens for completing educational modules, participating in their first five votes, and successfully submitting their first proposal. This program aims to lower the barrier to entry and foster a more inclusive and active governance community.

Algorithm 5 **Governance and Identity Management Primitives**

1: **Init:** *Q* (Quorum threshold)

2: **function** voteDAO(id,s)

3:    w←(tokens×%)/100

4:   proposal.votes +=s?w:−w

5: **end function**

6: **function** execDAO(id)

7:   **if** proposal.votes ≥Q **then** ▹ Vote weight exceeds quorum

8:    **require** treasury ≥ proposal.amt ▹ Check funds

9:    treasury −= proposal.amt

10:    payproposal.rcv, proposal.amt(proposal.rcv, proposal.amt)

11:   **end if**

12: **end function**

13: **function** issueSBT(rcv,uri)

14:    tid← tokenCtr

15:   mint(rcv,tid)

16:   setURI(tid,uri)

17:   soulbound[*tid*] = true

18:   tokenCtr++

19: **end function**

A key innovation of PolyMed is the integration of a **Decentralized Finance (DeFi)** module to address the economic barriers to care. The “DeFiLoanSC” smart contract establishes an on-chain protocol for undercollateralized microloans. The capital for these loans is sourced from **liquidity pools**, where any member of the community can stake their POL tokens to provide liquidity and, in return, earn a passive yield generated from the interest paid on loans. This creates a self-sustaining, community-funded financial engine within the platform. This model algorithmically encourages good repayment behavior and adapts to the broader DeFi market.

Since these are microloans, they are **undercollateralized**. Instead of requiring a patient to lock up significant capital, the system leverages their on-chain identity and reputation—represented by their SBT—as a form of social collateral. Should a user default on a loan, this event is permanently recorded on-chain, which can be used by the protocol to restrict future access to financial services. Key economic parameters, such as the maximum loan amount, interest rate, and staking APR, are established as on-chain variables that can be modified via DAO governance, allowing the DAO to adjust them in response to market conditions and community consensus. This ensures the long-term economic sustainability of the protocol.

**Smart Contract Security Framework:** Recognizing the inherent risks of DeFi protocols, the PolyMed system implements a three-tier security architecture: (1) Pre-deployment automated static analysis via Slither and manual audits by certified firms, (2) Runtime circuit breakers triggering automatic pauses when loan defaults exceed 15% in 24 h, and (3) Post-incident 48-h time-locked emergency functions requiring 67% DAO consensus. All critical administrative functions are placed under time-locks, giving the community time to review and veto changes through DAO votes.

**Economic Attack Prevention:** Flash loan protection is achieved through minimum 24-h holding periods before loan eligibility. To mitigate risks such as flash loan-based price manipulation, oracle resistance is achieved by sourcing data from a reputable, decentralized provider like Chainlink and using its 5-min time-weighted average pricing (TWAP) feeds, with on-chain logic to revert transactions if prices deviate more than 3% from the last known value. Liquidity drain protection caps individual withdrawals at 10% of total pool value per 24-h period.

**Emergency Procedures:** Critical failure scenarios trigger automated responses: Oracle failure activates fallback to secondary price feeds, smart contract exploits trigger immediate fund migration to predetermined safe contracts, and governance attacks activate emergency pause by any of 5 pre-designated multisig wallets. The logic for this module is formalized in Algorithm 6.

Algorithm 6 **DeFi Microloan Workflow Logic**

1:      ▹ System parameters, governable by DAO

2: **Init:** *u* (user), *t* (treasury), amt (amount), loanDB, stakeDB

3: **Constants:** MAX_LOAN = 500, INTEREST_BPS = 500,   STAKING_APR_BPS = 1,200, LOAN_COOLDOWN = 86,400

4: **function** requestLoan(*amt*)

5:   **require**(verified[u], “User not verified”);

6:   **require**(!loanDB[u].active, “Loan already active”);

7:   **require**(amt≤MAX_LOAN, “Amount exceeds max loan”);

8:   **require**(**NOW** -loanDB[u].repaidTimestamp ¿ = LOAN_    COOLDOWN, “Cooldown period active”);

9:    loanDB[u]← {principal: amt, repaid: 0, loanTimestamp:     **NOW**, repaidTimestamp: 0, active: **true**}

10:   POL.transfer(u,amt)

11: **end function**

12: **function** repayLoan(amt)

13:   **require**(loanDB[u].active, “No active loan”);

14:   POL.transferFrom(u,t,amt)

15:    loanDB[u].repaid += *amt*

16:   interestOwed ←loanDB[u].principal * INTEREST_      BPS/10,000;

17:   **if** loanDB[u].repaid ≥loanDB[u].principal + interestOwed     **then**

18:     loanDB[u].active ← **false**;

19:     loanDB[u].repaidTimestamp ← **NOW**;

20:   **end if**

21: **end function**

22: **function** stake(*amt*)

23:   **require**(verified[u], “User not verified”);

24:   **require**(amt>0, “Cannot stake zero”);

25:   POL.transferFrom(u,t,amt)

26:   stakeDB[*u*].amount += *amt*;

27:   stakeDB[*u*].timestamp ← **NOW**;

28: **end function**

29: **function** unstake()

30:    s←stakeDB[*u*];

31:   **require**(s.amount>0, “No funds staked”);

32:       ▹ Note: This APR is likely unsustainable if higher       than loan interest.

33:   elapsedTime ← **NOW** -s.timestamp;

34:   rewards      ←(s.amount∗STAKING_APR_BPS/      10,000elapsedTime)/(365∗86,400);

35:   POL.transfer(u,s.amount+rewards)

36:   stakeDB[*u*] ← **null**

37: **end function**

## 4 Performance analysis and scalability

To validate the PolyMed architecture and assess its viability for real-world clinical deployment, a rigorous performance evaluation was conducted. The evaluation was designed to be comprehensive, focusing on two critical areas: first, the efficiency, cost, and scalability of the foundational on-chain operations, and second, the predictive accuracy and reliability of the AI-driven clinical intelligence module. This section details the evaluation methodology, presents the empirical results from the benchmarking tests, and provides a detailed validation of the AI model's performance.

### 4.1 On-chain performance benchmarking

The evaluation of the blockchain backbone of PolyMed was conducted on the **Polygon Mainnet**. This choice was deliberate to ensure that the results reflect real-world network conditions, including variable gas prices and network congestion, rather than the idealized conditions of a local testnet. This approach provides a much more accurate assessment of the system's operational performance.

To ensure the replicability and transparency of the findings, the full system configuration used for benchmarking is detailed in Table 3. This standardized testing environment was employed consistently across all empirical evaluations presented in this paper. Core smart contract functions that represent the most common interactions within the PolyMed ecosystem were selected: EHR Upload, Record Retrieval, Appointment Scheduling, Prescription Issuance, Doctor Verification, DAO Proposal Submission, and Token-Weighted Voting. The statistical analysis in this study is primarily descriptive, and the AI model evaluation employs standard performance metrics common in the machine learning domain. To generate a realistic transactional load and to ensure the results were statistically significant, each of these operations was executed 500 times. The load was generated by 30 concurrent clients, a number chosen to simulate the activity of a small-to-medium-sized clinic. The clients were run on virtual machines and orchestrated using a custom test script built with the Hardhat and Ethers.js, interacting with the Polygon Mainnet via an Infura API endpoint. Average latency and gas consumption were recorded for each operation type.

**Table 3:** Standardized system configuration used for All performance evaluation benchmarks.

Component	Specification
Blockchain network	Polygon mainnet
Client machines	30 virtual machines (2 vCPU, 4 GB RAM)
Web3 provider	Infura API
Client wallet	MetaMask (automated via Ethers.js)
Test framework	Hardhat, chai
Network connection	1 Gbps fiber optic

The performance results, summarized in Table 4, demonstrate the high efficiency of the PolyMed system on the Polygon network. All core operations achieved average transaction confirmation latencies of under 4 s. This is a critical finding, as this sub-4-second response time directly improves clinical workflows by meeting the demand for near-instant data access during patient consultations or emergencies. Furthermore, the average gas costs were minimal, highlighting the platform's economic viability.

**Table 4:** Average transaction latency and Gas consumption for core system operations, measured on the polygon mainnet (*N* = 500 runs per operation).

Operation	Avg. latency (s)	Avg. gas (POL)
EHR upload	3.87	0.0125
Record retrieval	2.16	0.0073
Appointment scheduling	2.91	0.0097
DAO proposal submission	3.44	0.0157
Token-weighted voting	3.02	0.0113
Prescription issuance	2.78	0.0107

**Figure 3:**
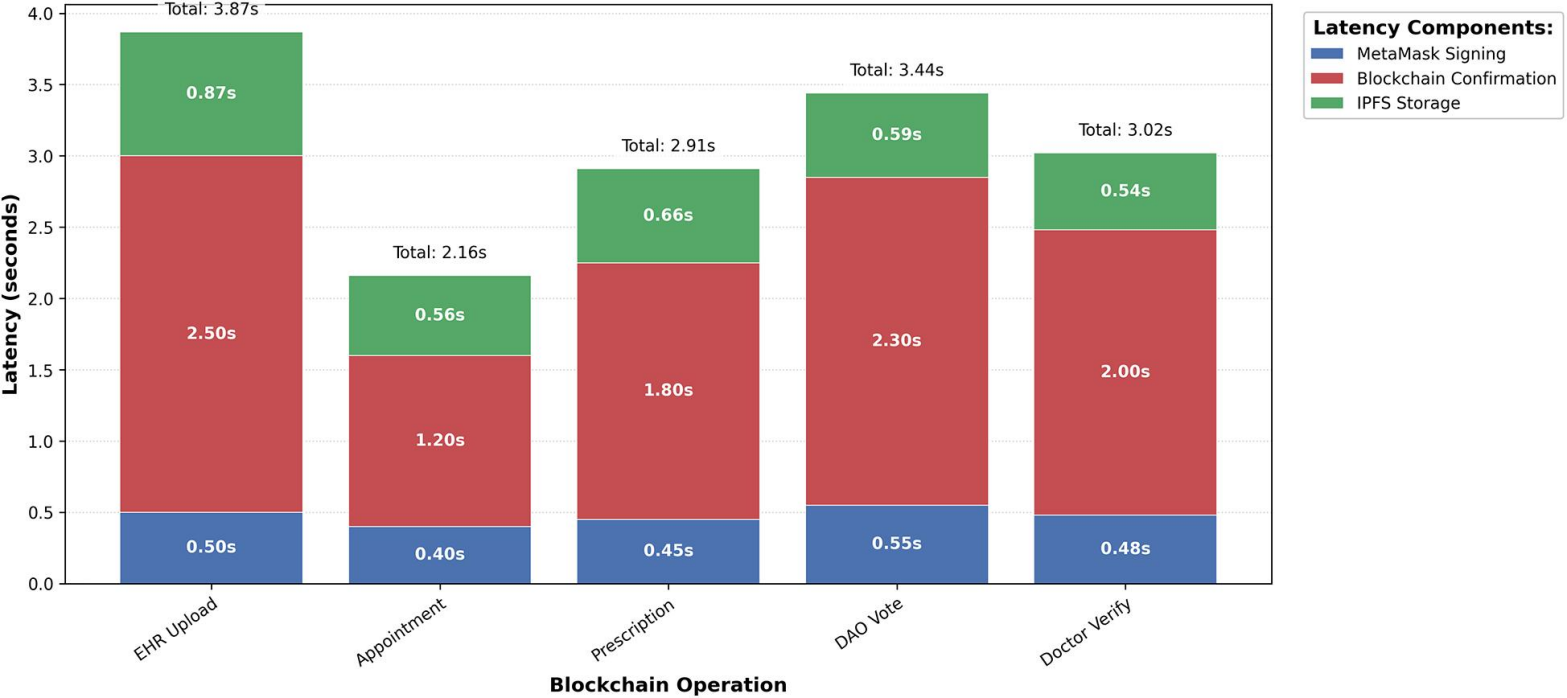
Latency composition analysis by operation. This chart dissects the total average latency for each transaction type, with the aggregate values detailed in Table 4. The visualization highlights that on-chain confirmation is the most time-consuming step, whereas client-side signing and off-chain storage operations are significantly faster.

To provide a more granular analysis of these latency results, Figure 3 breaks down the total transaction time for each core operation into its primary components. This composition analysis reveals that blockchain confirmation constitutes the largest portion of the latency, which is an expected characteristic of the underlying network's consensus mechanism. The contributions from MetaMask signing and IPFS storage are comparatively minor, confirming that the on-chain validation step is the main determinant of overall response time.

**Figure 4:**
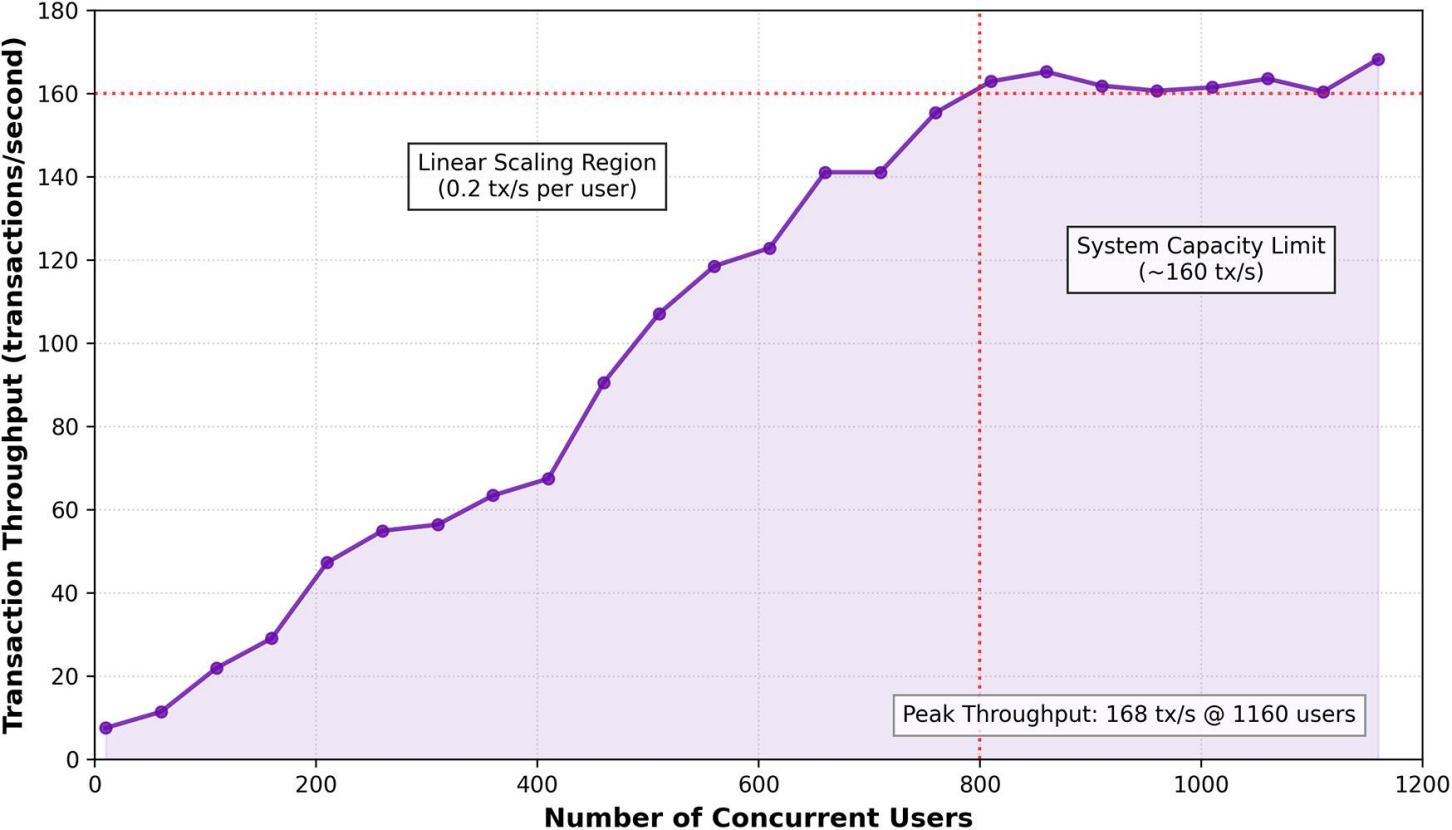
Transaction throughput vs. Concurrent Users. The system demonstrates linear scaling up to approximately 800 concurrent users, reaching a peak throughput of over 160 transactions per second. This confirms the platform's ability to handle the load of a medium-to-large clinical environment, with current limitations defined by off-chain dependencies like public IPFS gateways.

**Figure 5:**
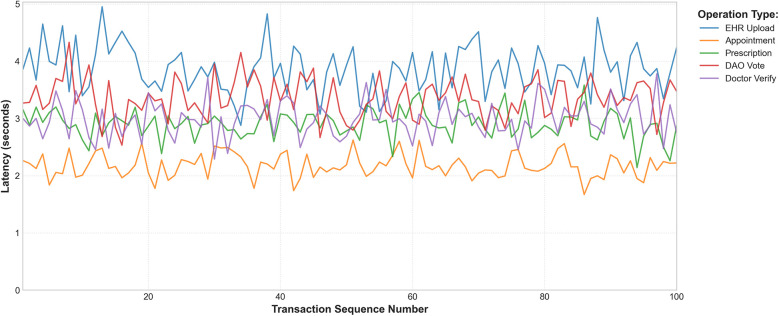
Blockchain operation latency trends (100 consecutive transactions). This line graph illustrates the latency for a sequence of 100 transactions for each major operation. The stable, bounded fluctuations visually confirm the system's consistent performance over time, a key requirement for real-world blockchain applications.

To assess the system's ability to scale, a comprehensive evaluation was conducted using the experimental setup detailed in Section 4.1. The throughput test, scaling from 30 to 800 concurrent clients, is illustrated in Figure 4. The system maintained stable throughput up to approximately 800 concurrent users, beyond which latency increased due to rate-limiting at the public IPFS gateway. This result is consistent with findings on scalable blockchain architectures for healthcare (15). Operational resilience, tested under various fault conditions (Table 5), proved to be robust, with high success rates supported by effective fallback mechanisms. The consistency of the system's performance is further demonstrated in Figure 5, which plots the latency for 100 consecutive transactions for each core operation. Despite natural network fluctuations, the latencies remain within a stable and predictable range, affirming the platform's operational reliability under a sustained transactional load.

**Table 5:** System resilience under Various simulated fault conditions, showing high success rates and effective fallback mechanisms.

Scenario	Success rate	Fallback mechanism
Polygon Congestion	98.3%	Client-side Delayed Retry
IPFS Partial Failure	95.6%	Redundant Gateway Connection
MetaMask Disconnect	93.1%	Session Re-authentication
DAO Vote Timeout	100%	Commit-Reveal Logic (a two-stage voting process to prevent vote influence)

### 4.2 AI Model performance validation

The clinical viability of the PolyMed platform is critically dependent on the reliability of its AI-driven emergency detection module. To ensure a robust evaluation, a 10-fold stratified cross-validation methodology was employed, and the proposed LightGBM model was rigorously benchmarked against a standard Logistic Regression baseline.

The emergency detection module was trained on the public **PhysioNet/Computing in Cardiology Challenge 2012 dataset** (21). An advanced feature engineering pipeline was used to transform raw time-series data into a rich tabular feature set by calculating statistical moments and trend slopes across multiple time windows (6, 12, 24, and 48 h). A **LightGBM (LGBM)** classifier was trained on this feature set. To handle the significant class imbalance, the “scale_pos_weight” parameter was utilized. Hyperparameters were tuned using the Optuna framework over 50 trials, with a 10-fold cross-validation strategy inside each trial. The final optimized hyperparameters are shown in Table 6.

**Table 6:** Optimized hyperparameters for the LightGBM model.

Hyperparameter	Optimized value
n_estimators	1,200
learning_rate	0.0364
num_leaves	90
max_depth	14
subsample	0.8557
colsample_bytree	0.8009

**Figure 6:**
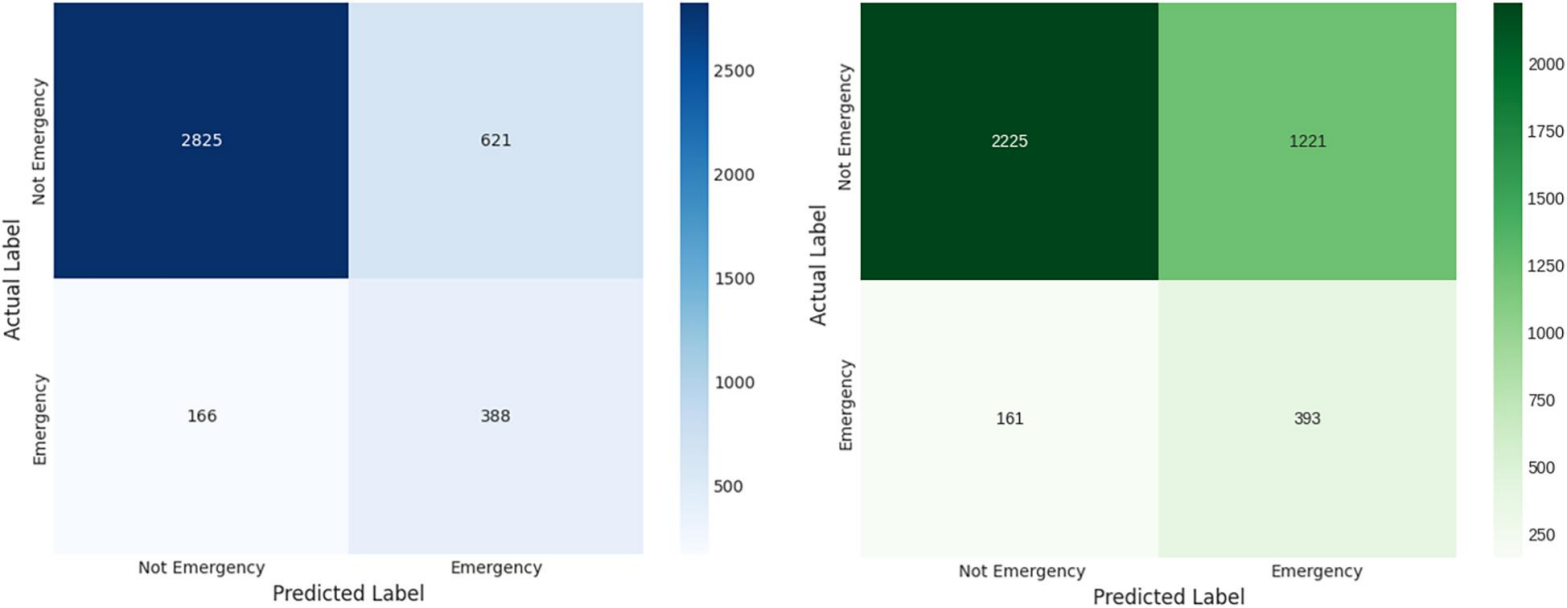
Aggregated confusion matrices for (left) the proposed LGBM model and (right) the logistic regression baseline. These matrices show the summed predictions across all 10 folds, providing a clear view of each model's error profile. The LGBM model demonstrates a superior balance, committing significantly fewer False Positive errors (incorrectly flagged emergencies) while successfully identifying the majority of True Positives (correctly identified emergencies).

The final performance of both models, averaged across the 10 folds, is detailed in Table 7. A key clinical requirement was to minimize missed emergencies, so both models were calibrated to achieve a high-sensitivity operating point targeting a minimum recall of 70%. The results show that the proposed LGBM model significantly outperforms the baseline across all other key metrics. A visual comparison of the models' error types is presented in the aggregated confusion matrices in Figure 6.

**Table 7:** Ai model performance comparison (mean ± Std. Dev.) from 10-fold Cross-Validation.

Metric	LGBM (proposed)	Logistic regression (baseline)
Accuracy	80.33% ± 1.56%	65.45% ± 5.26%
AUC	0.8543 ± 0.0099	0.7389 ± 0.0230
Precision	38.60% ± 2.43%	24.80% ± 3.49%
Recall	70.04% ± 1.65%	70.93% ± 2.60%
F1-Score	0.4972 ± 0.0202	0.3660 ± 0.0369

**Figure 7:**
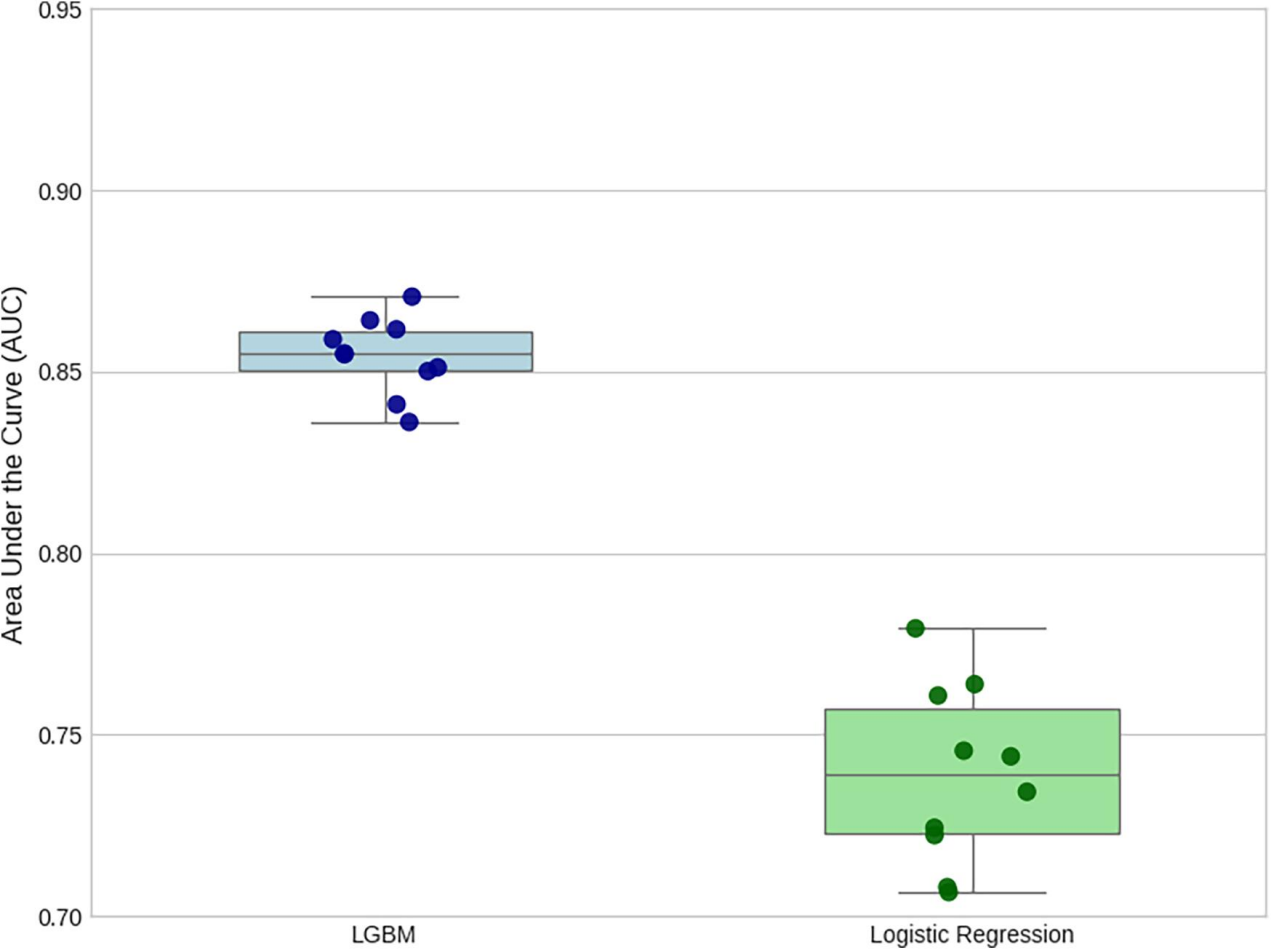
Comparison of AUC score distributions from 10-Fold cross-validation. This plot visualizes the performance and stability of each model across the 10 folds. The LGBM model exhibits both a higher median AUC and a much tighter interquartile range, indicating that it is not only more accurate on average but also significantly more consistent in its performance than the baseline.

**Figure 8:**
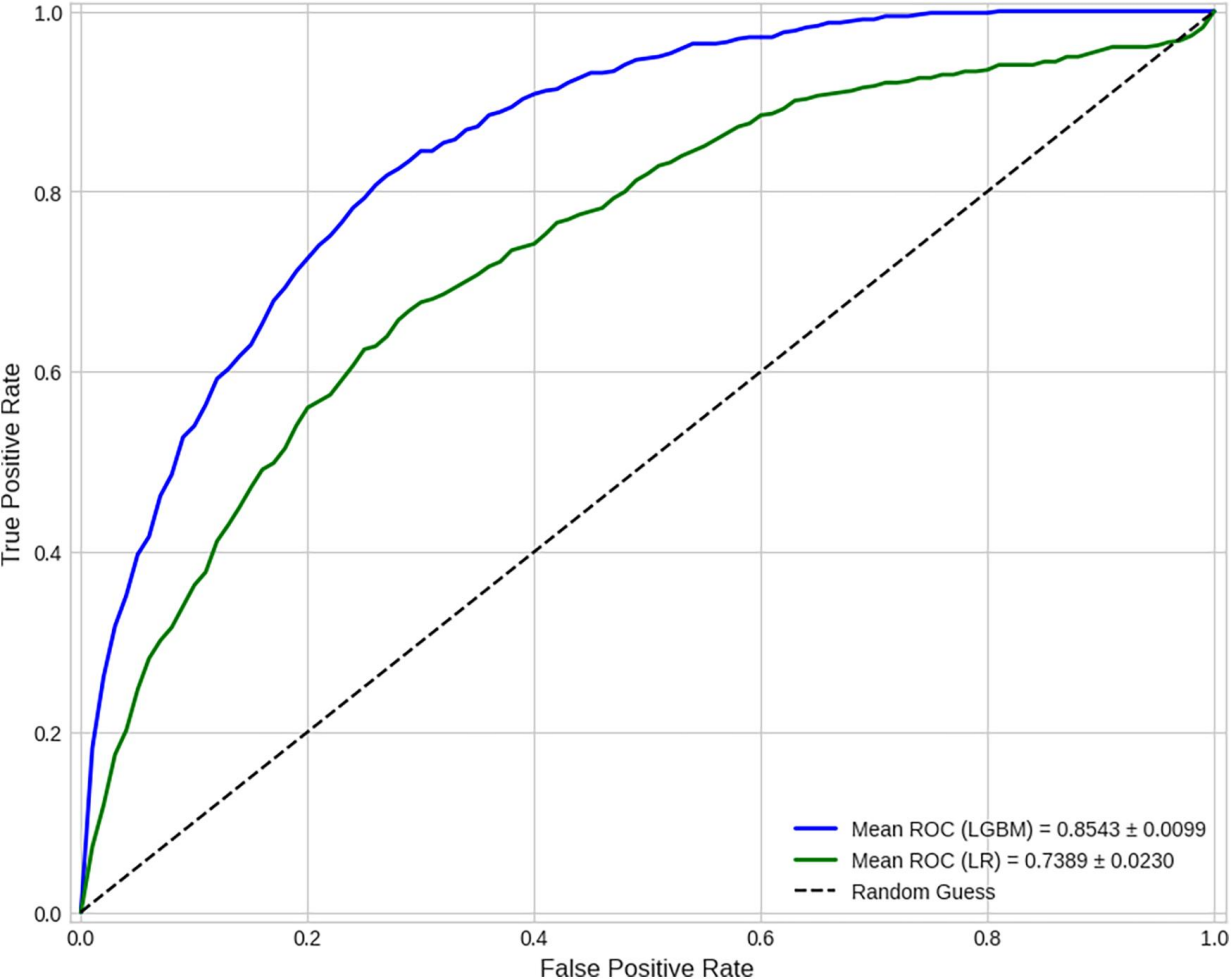
Comparative ROC curves (averaged over 10 folds). This curve illustrates a model's ability to distinguish between classes across all classification thresholds. The mean Area Under the Curve (AUC) of **0.8543** for the LGBM model demonstrates its strong and superior discriminative power compared to the baseline model's AUC of 0.7389.

**Figure 9:**
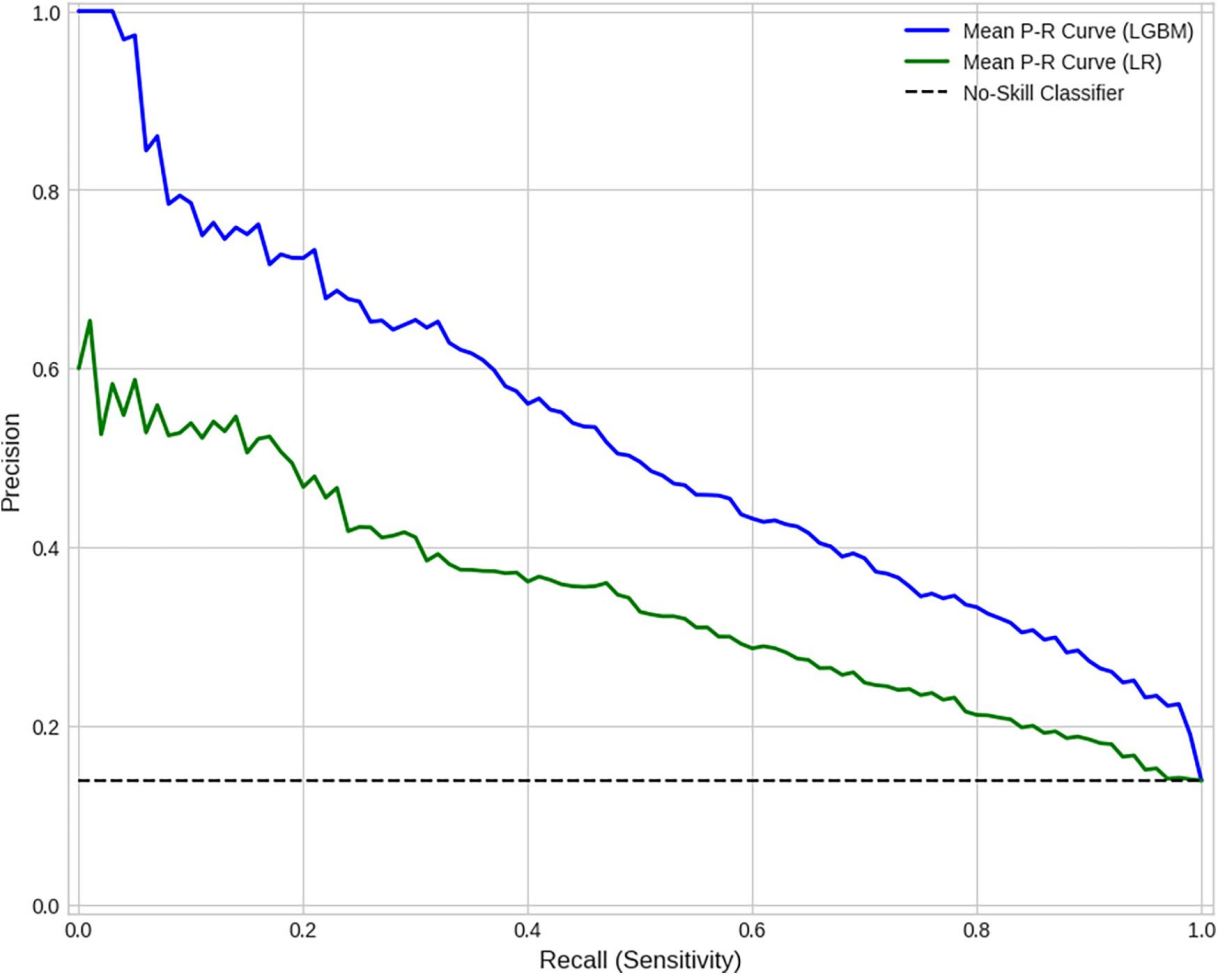
Comparative precision-recall curves (averaged over 10 folds). This curve is particularly insightful for imbalanced datasets as it visualizes the trade-off between a model's Precision and Recall on the positive class. For any given level of recall (sensitivity), the LGBM model achieves substantially higher precision than the baseline, highlighting its superior predictive accuracy for identifying “Emergency” events.

The superior discriminative power of the LGBM model is further illustrated by the comparative ROC curve in Figure 8, which shows a substantially higher Mean AUC. The stability of this performance is confirmed by the comparative box plot in Figure 7, which visualizes the distribution of AUC scores from each of the 10 folds. Finally, the comparative Precision-Recall curve in Figure 9 is particularly insightful for this imbalanced dataset, demonstrating that for any given level of recall, the LGBM model maintains significantly higher precision.

To formally prove that the observed performance difference between the LGBM classifier and the Logistic Regression baseline was not due to chance, a suite of complementary statistical tests was performed. A Logistic Regression model was chosen as the baseline because it is a robust, well-established, and highly interpretable industry standard for binary classification.

First, a paired *t*-test was chosen to compare the means of the two related samples (LGBM and baseline scores) generated from the 10-fold cross-validation. This test confirmed a statistically significant advantage for the LGBM model [***t*(9)** **=** **16.20, *p*** < **0.001**]. To confirm this finding without relying on the assumption of normal distribution, the non-parametric Wilcoxon signed-rank test was also applied as a robust alternative. This test corroborated the *t*-test's conclusion, likewise indicating a statistically significant improvement (***W*** **=** **0.0, *p*** **=** **0.002**).

Finally, to determine if there was a significant difference in the types of errors made by the two models, McNemar's test was performed, as it is specifically designed for comparing paired nominal data from two classifiers. This test is specifically designed to assess the significance of the difference between two classifiers by analyzing the discordant pairs (cases where one model was correct and the other was incorrect). As shown in the contingency data in Table 8, the proposed LGBM model made significantly fewer errors than the baseline on these discordant cases (834 vs. 239). The resulting Chi-squared statistic, calculated from the discordant pairs where the models disagreed (834 vs. 239), confirms that this difference in error rates is highly significant $\left(\chi^{2}(1)=328.8,p<0.001\right)$. Taken together, these three tests provide converging statistical evidence that the LGBM classifier delivers a robust and practically meaningful improvement over the baseline.

**Table 8:** Contingency table for mcNemar's test, showing prediction agreement and disagreement between the models over 4,000 aggregated samples.

Outcome	Baseline correct	Baseline incorrect
LGBM correct	2,379	834
LGBM incorrect	239	548

## 5 Security and compliance frameworks

PolyMed places a paramount emphasis on security and regulatory compliance, adopting a “privacy-by-design” approach that is explicitly aligned with leading global mandates governing personal health data. This commitment is demonstrated through formal threat modeling and a comprehensive compliance analysis.

### 5.1 Threat modeling and mitigation

A formal threat analysis was conducted using the STRIDE framework (Spoofing, Tampering, Repudiation, Information Disclosure, Denial of Service, and Elevation of Privilege) to ensure system-wide resilience. The analysis systematically identified potential threats and outlined corresponding mitigation strategies, detailed in Table 9. These mitigations leverage the specific architectural components detailed in the methodology, such as ZKPs for spoofing resistance and on-chain immutability for tamper evidence. PolyMed's security is further hardened by a suite of industry-standard cryptographic primitives, as detailed in Table 10, ensuring data confidentiality, integrity, and authenticity at every stage.

**Table 9:** STRIDE threat analysis and mitigation strategies for the PolyMed system.

Type	Threat description	Likelihood	Impact	Mitigation strategy
S	Identity Spoofing via forged credentials	High	High	Multi-factor authentication using MetaMask wallet signatures combined with the ZKP-based Aadhaar verification workflow detailed in Section 3. Non-transferable Soulbound Tokens (SBTs) bind verified identities to specific wallets, preventing credential reuse.
T	Tampering with on-chain/off-chain health data	Medium	High	On-chain immutability of record hashes prevents tampering with pointers. Off-chain data is end-to-end encrypted (AES-256) and its integrity is verifiable against the on-chain hash, as per the IoMT pipeline design.
R	Transaction repudiation by malicious actors	Low	Medium	All transactions are cryptographically signed and permanently recorded on the Polygon blockchain, creating a non-repudiable audit trail of all actions, including access grants and data modifications.
I	Information disclosure through unauthorized access	Medium	High	Fine-grained, role-based access control is enforced by smart contracts that check for specific SBTs. ZKP-based queries are architected to enable aggregate analytics without revealing individual records.
D	Denial-of-Service attacks targeting APIs and chains	Medium	Medium	The decentralized nature of the Polygon validator set provides high resilience. Rate limiting on API endpoints and the use of multiple redundant IPFS gateways further mitigate risks.
E	Unauthorized privilege escalation	Low	High	Access privileges are programmatically tied to non-transferable SBTs. Any change in a user's role or core system permissions must be approved via a formal DAO proposal, requiring community consensus.

**Table 10:** Cryptographic primitives and their applications in PolyMed.

Primitive	Application	Purpose	Security strength (bits)
SHA-256	Hashing EHR data, transaction IDs, Merkle roots	Data integrity, tamper detection, unique identification	256
AES-256	End-to-end encryption of off-chain EHR data	Data confidentiality, privacy protection	256
ECDSA	MetaMask wallet signatures, transaction signing	User authentication, transaction authenticity, non-repudiation	256 (eq. to 3,072-bit RSA)
ZK-SNARKs	Anon-Aadhaar identity verification, privacy-preserving queries	Identity verification without revealing PII, anonymous data analytics	Varies, typically >128
Merkle Trees	Transaction audits, data integrity verification in IPFS	Efficient verification of data integrity and consistency (akin to a digital table of contents for data blocks)	Logarithmic in data size

**Figure 10:**
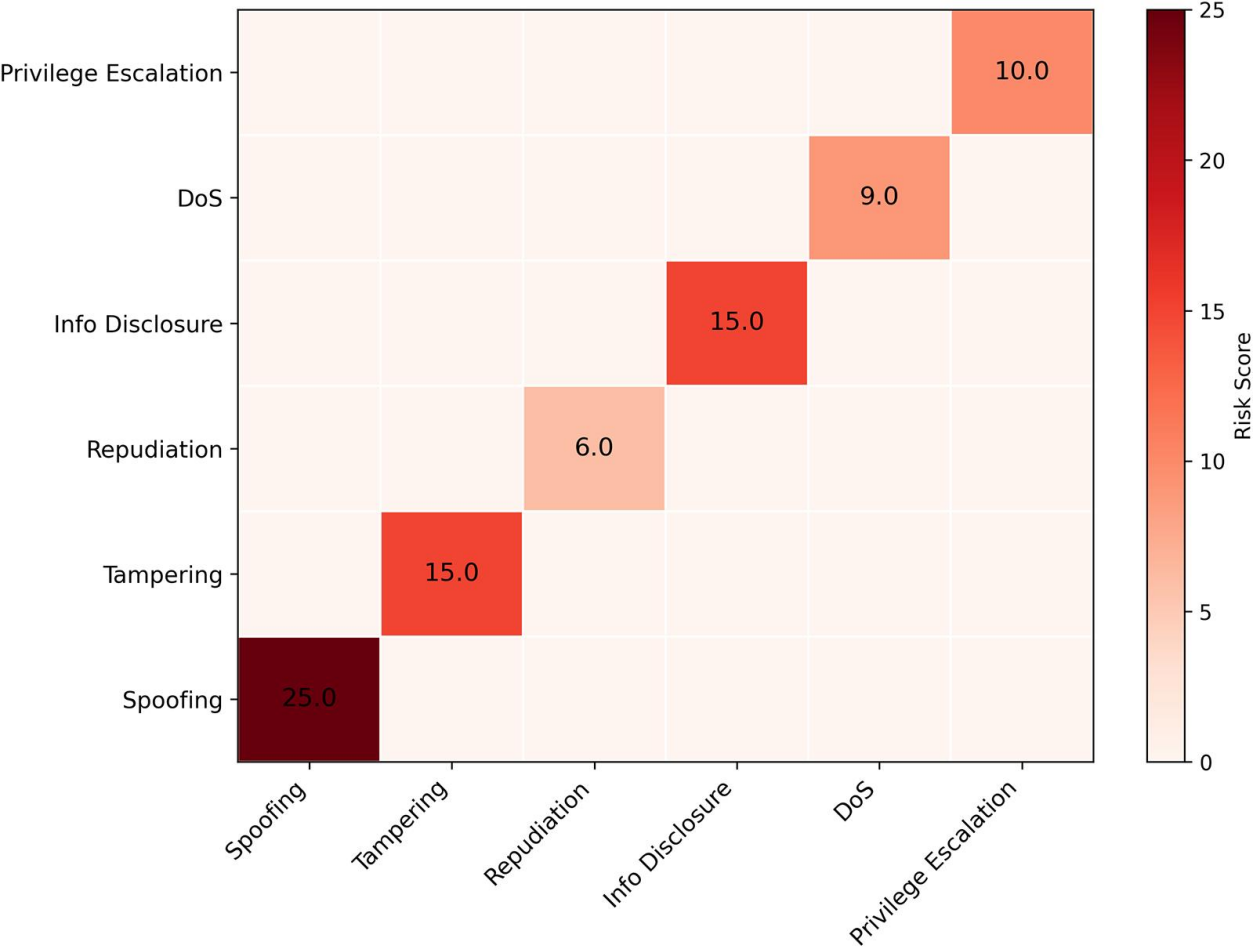
Residual risk heatmap. This heatmap visualizes the residual risk for each STRIDE category after mitigation strategies are applied, confirming that identity and tampering risks, while high in impact, are significantly mitigated by the system's multi-layered cryptographic design.

The residual risk, visualized in the heatmap in Figure 10, highlights identity spoofing and data tampering as the highest initial risk areas, confirming that PolyMed's cryptographic safeguards are appropriately focused on the most critical vulnerabilities. While not experimentally computed in this study, the overall system security score, $S_{\mathrm{sys}}$, can be formally modeled as a weighted average of the inverse of these residual risks, providing a framework for future quantitative security audits:

(9) $$S_{\mathrm{sys}}=\frac{\sum_{N_{\mathrm{threats}}}^{i=1}w_{i}/\mathrm{Ris}k_{i}}{\sum_{N_{\mathrm{threats}}}^{i=1}w_{i}}$$

### 5.2 Regulatory and compliance alignment

**Figure 11:**
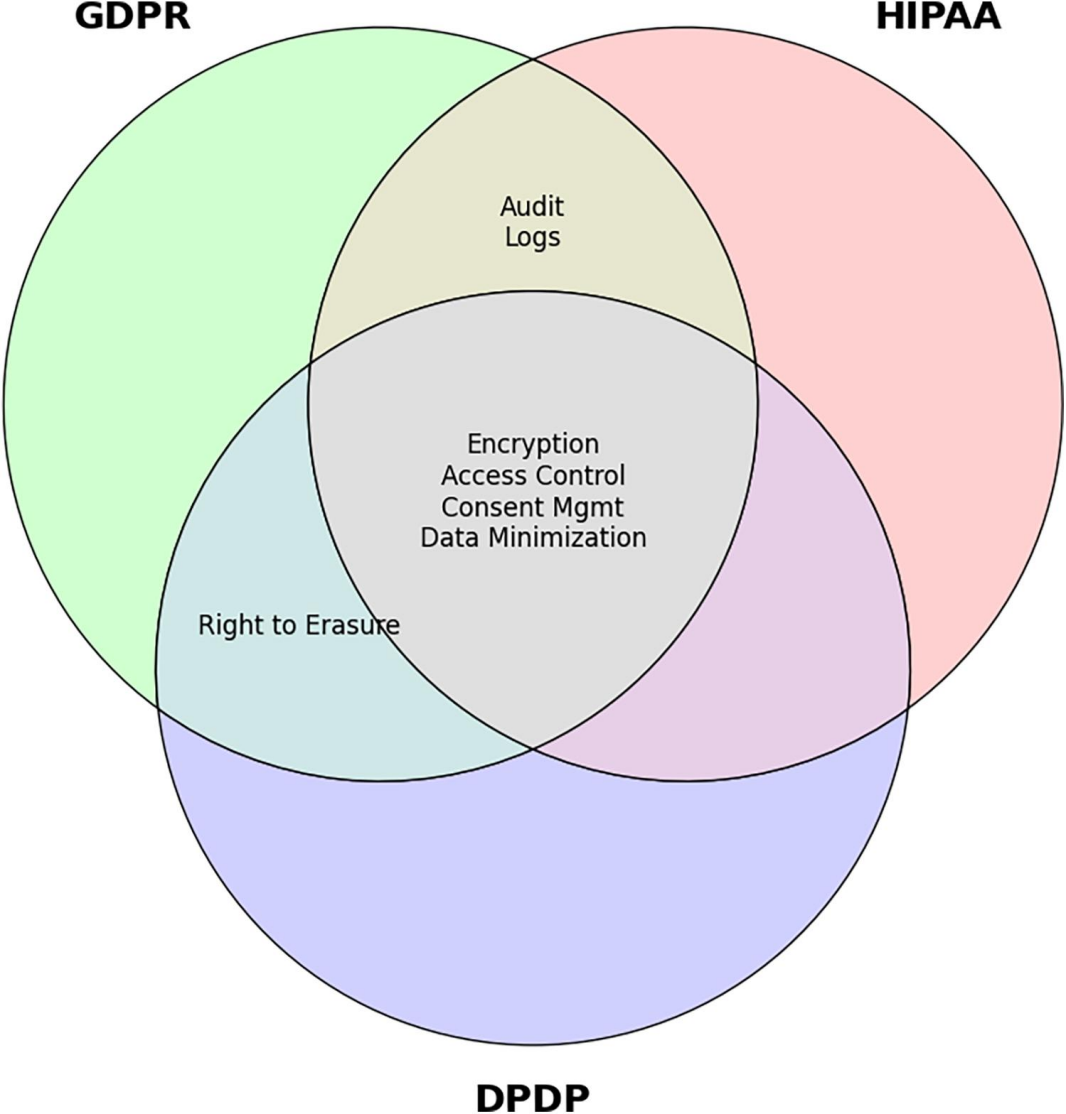
Venn diagram of overlapping compliance features. This diagram visually illustrates the common and distinct requirements of GDPR, HIPAA, and India's DPDP Act, highlighting PolyMed's ability to address shared mandates like encryption and access control.

PolyMed is developed to align with core provisions of three leading regulatory frameworks: GDPR, HIPAA, and India's Digital Personal Data Protection (DPDP) Act. The compliance matrix in Table 11 details these features. A key challenge is reconciling the “right to erasure”—a right guaranteed under regulations like GDPR and India's DPDP Act—with the technical immutability of a blockchain. PolyMed resolves this via a cryptographic “soft delete” mechanism: the off-chain encrypted data is permanently deleted, and a DAO-approved transaction disassociates its on-chain link from the patient's identity. This approach functionally fulfills the erasure request without altering blockchain history, a recognized best practice for decentralized systems. This targeted solution is part of a broader compliance framework, detailed in Table 11, which maps system features to specific regulatory requirements. The Venn diagram in Figure 11 visually summarizes this multi-jurisdictional alignment, illustrating how core features like encryption and access control satisfy the overlapping mandates of all three frameworks, while other features address specific regional laws.

**Table 11:** Regulatory compliance feature matrix for PolyMed.

Compliance feature	GDPR	HIPAA	DPDP act (IN)
Consent-based access	✓	✓	✓
Right to erasure (soft delete)	✓	✗	✓
End-to-end encryption	✓	✓	✓
Role-based access control	✓	✓	✓
Tamper-proof audit trails	✓	✓	✓
Pseudonymization (ZK proofs)	✓	✗	✓
Data portability (IPFS link)	✓	✗	✓

## 6 Usability and economic implications

Beyond technical and security validation, real-world viability depends on user experience and economic sustainability.

### 6.1 Usability study

To gather initial feedback on the platform's design, a preliminary usability study was conducted with 20 participants (10 medical students, 5 licensed clinicians, 5 non-technical administrators). Participants performed core tasks and were evaluated using two primary instruments: a 5-point Likert scale for specific usability heuristics (summarized in Table 12) and the standardized System Usability Scale (SUS) questionnaire for a holistic measure of user satisfaction.

**Table 12:** User perception scores on PolyMed's usability heuristics (1–5 scale, *N* = 20). All scores were statistically significant (*p* < 0.01) compared to a neutral midpoint of 3.0.

Usability metric	Mean score	Std. dev.	95% CI
Learnability	4.6	0.50	[4.37, 4.83]
Efficiency	4.3	0.66	[3.99, 4.61]
Memorability	4.5	0.51	[4.26, 4.74]
Error Prevention	4.7	0.47	[4.48, 4.92]
Satisfaction	4.8	0.41	[4.61, 4.99]

The SUS questionnaire captures a spectrum of user perceptions through a series of 10 statements. To provide insight into the scope of the evaluation, these included items assessing desirability (“I think that I would like to use this system frequently”), perceived complexity (“I found the system unnecessarily complex”), and user confidence (“I felt very confident using the system”). The SUS score provides a composite measure of usability and can be formally calculated as:

(10) $$\mathrm{SUS}\;\mathrm{Score}=2.5\times \left(\sum_{10}^{i=1}\mathrm{TransformedScor}e_{i}\right)$$

where “TransformedScore” is derived from the user's Likert scale responses to the 10 SUS items.

**Figure 12:**
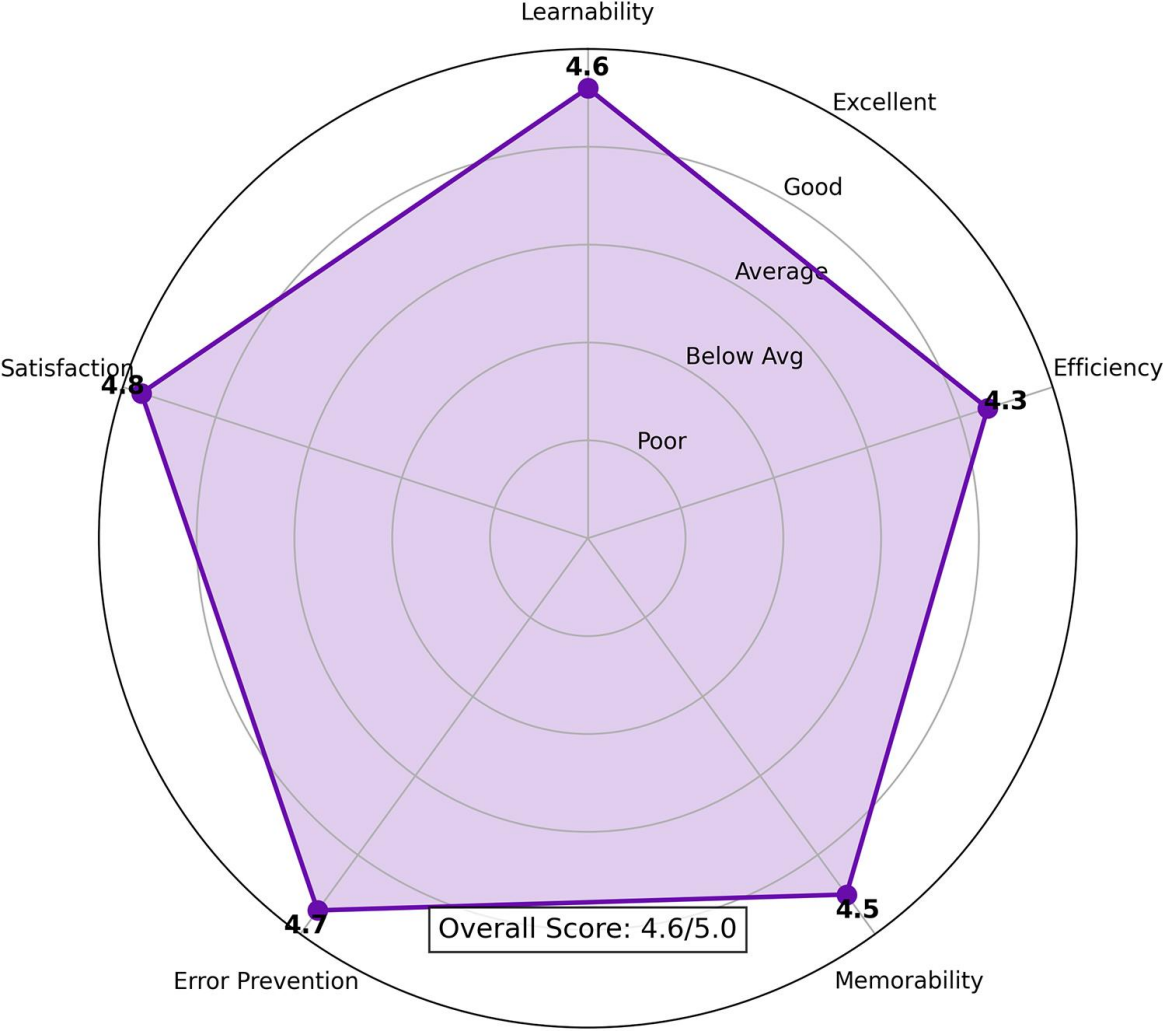
Polymed usability evaluation (*N* = 20). The radar chart visualizes the mean scores for key usability heuristics, showing strong performance across all categories. The detailed numerical scores, including statistical analysis, are presented in Table 7.

The quantitative results are summarized in Table 12. To satisfy the reviewer's call for more robust analysis, a formal statistical analysis was conducted. Descriptive statistics including mean, standard deviation (Std. Dev.), and 95% confidence intervals (CI), selected in accordance with scientific convention, were calculated from the raw scores of the 20 participants for each metric. The standard deviation measures the dispersion of participant responses, while the 95% CI provides an estimated range for the true mean of the broader user population. The CIs were calculated using the sample mean, the standard error, and a critical value derived from a standard *t*-distribution. The specific critical value is determined by the degrees of freedom (*n* − 1 = 19) and the chosen confidence level, making this the appropriate method for a small sample size (*N* = 20). Furthermore, a one-sample Wilcoxon signed-rank test was performed for each heuristic against a neutral midpoint of 3.0, confirming that all user scores were statistically significantly positive (*p* < 0.01). The platform excelled in **Satisfaction (4.8** ± **0.41)** and **Error Prevention (4.7** ± **0.47)**, indicating high user satisfaction and a low error rate. The radar chart in Figure 12 visualizes these strong results, culminating in a high overall average score of 4.6 out of 5.0.

**Figure 13:**
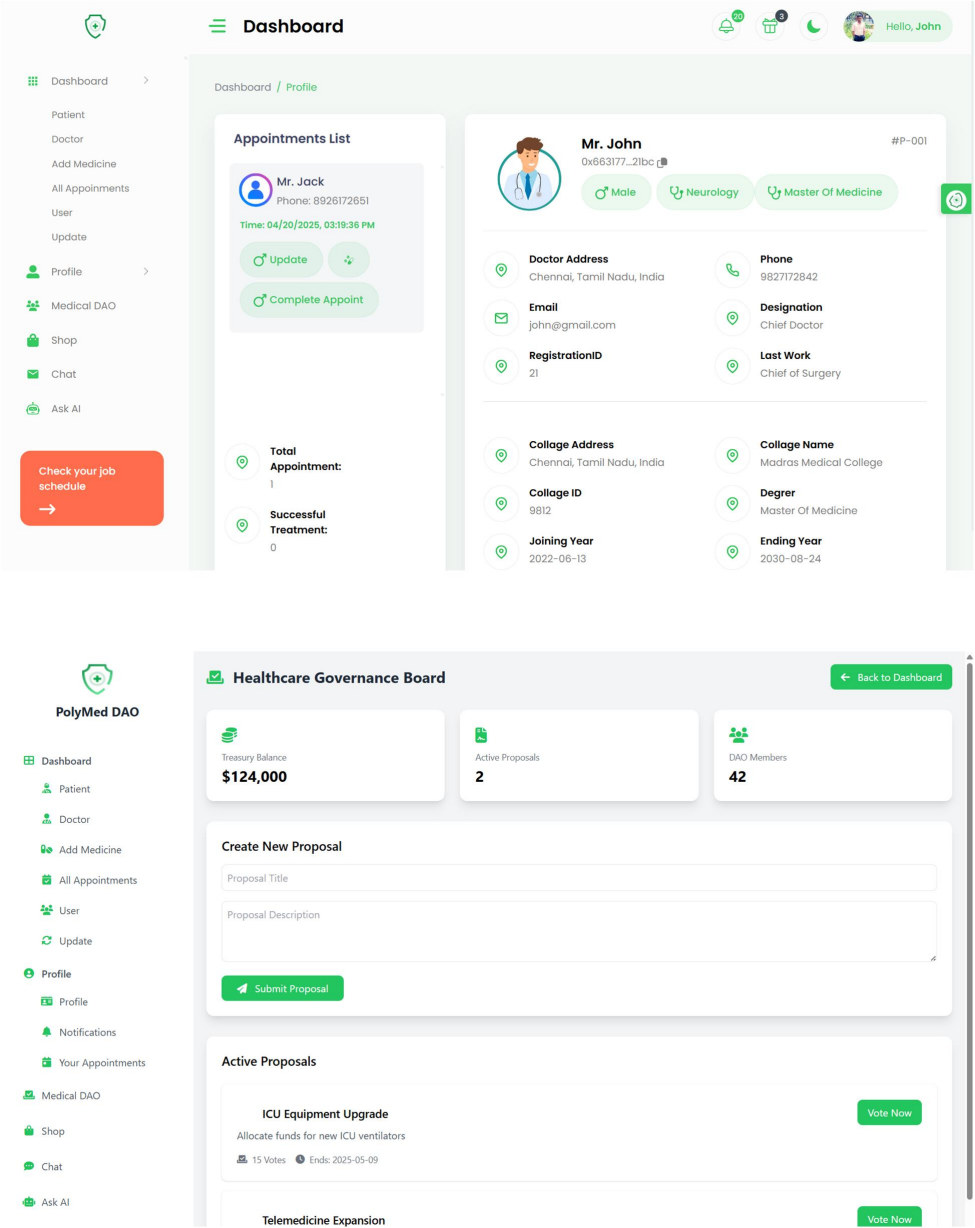
Polymed user interface mockups evaluated in the usability study. (Top) The main patient dashboard designed for intuitive management of health records. (Bottom) The portal for participating in DAO governance, which was identified as an area needing improved user onboarding.

Qualitative feedback was also positive, with participants highlighting the transparency of on-chain processes. However, several users noted a “**DAO Literacy Gap,”** indicating a need for better user onboarding for the governance module, a challenge addressed by the framework described in Section 3. This feedback aligns with the relatively lower score for **Efficiency (4.3** ± **0.66)**, which suggests that workflows around advanced features could be streamlined. The small sample size of this study is a significant limitation, and a larger, more diverse study including real patients is a key area for future work. Figure 13 shows mockups of the user interface evaluated in the study.

### 6.2 Economic analysis

**Figure 14:**
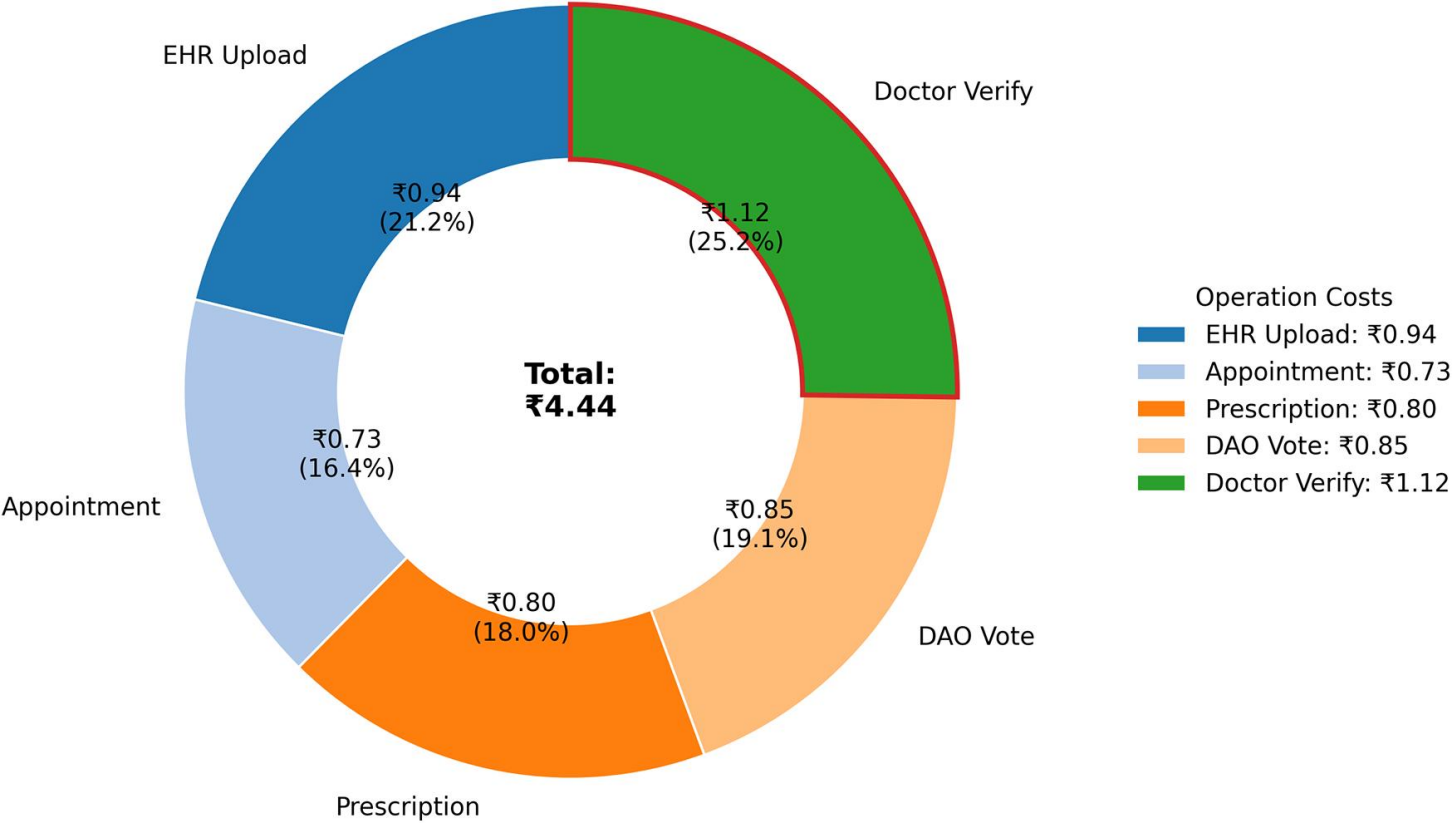
Relative Gas cost Per patient visit by operation (INR). This chart illustrates the breakdown of the total on-chain cost for a typical patient interaction. The precise cost and percentage for each operation are provided in Table 8.

PolyMed is engineered for affordability, leveraging Polygon's low transaction costs. Table 13 details the average cost of core operations in INR, demonstrating remarkable cost efficiency. The relative contribution of each operation to the total cost per visit is visualized in Figure 14.

**Table 13:** Gas Fee breakdown for core operations (rate: |75/POL, April 2024), highlighting the Low cost of on-chain interactions.

Operation	Avg. gas (POL)	Cost (INR)
EHR upload	0.0125	|0.94
Appointment scheduling	0.0097	|0.73
Prescription issuance	0.0107	|0.80
DAO voting	0.0113	|0.85
Doctor verification	0.0149	|1.12
**Cumulative (per visit)**	**0.0591**	**|4.44**

**Figure 15:**
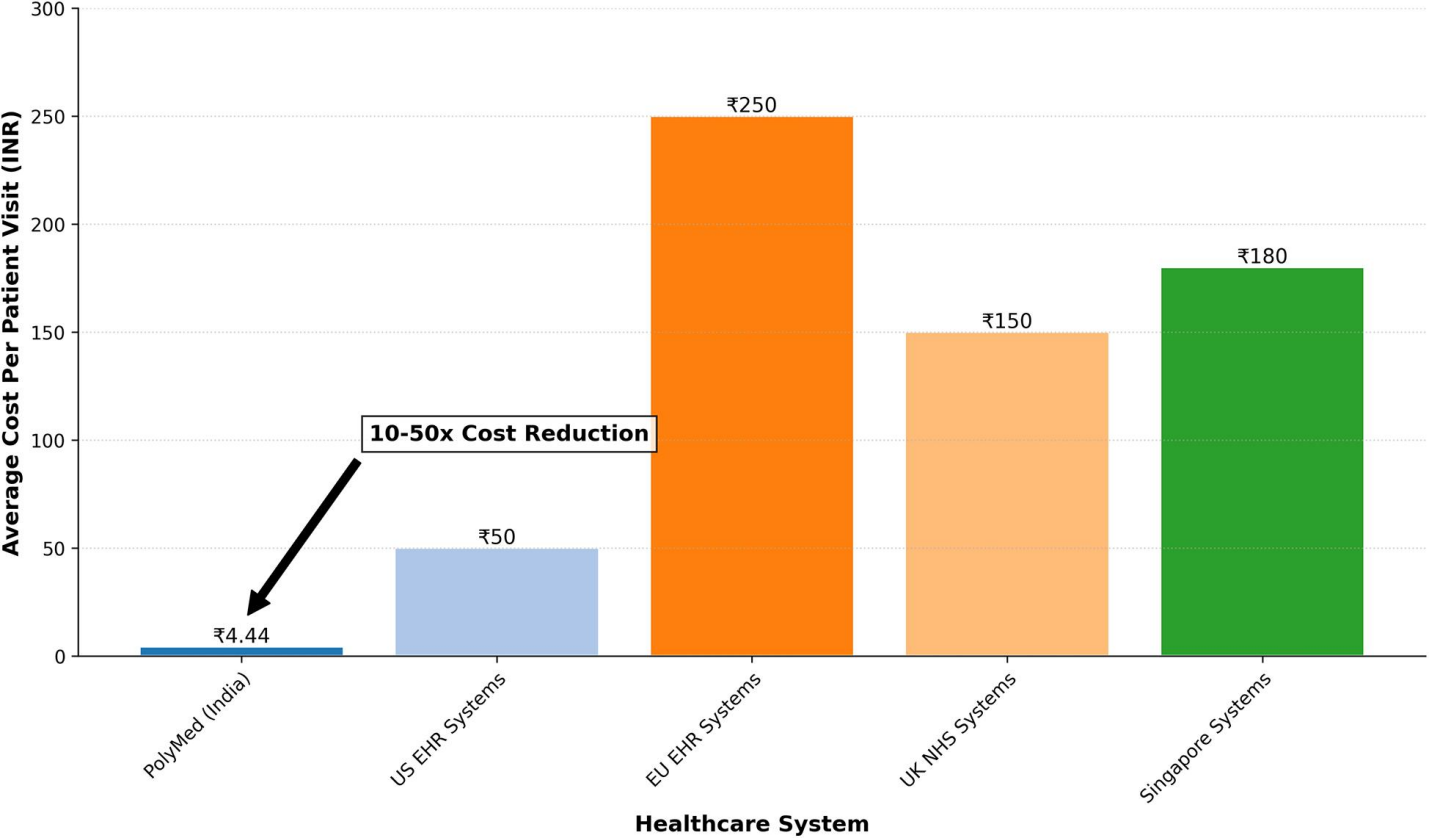
Cross-Country comparison of EHR interaction costs (per patient visit). This chart visualizes the dramatic cost efficiency of PolyMed's on-chain operations compared to the estimated operational costs of traditional EHR systems. The corresponding data is listed in Table 14.

Figure 15 provides a comparative analysis, detailed further in Table 14, showing that PolyMed offers substantial cost savings over traditional EHR systems. While a direct cost comparison with other Layer-2 blockchain solutions is complex, Polygon is consistently ranked as one of the most cost-effective platforms for high-volume transactions. The reliance on the POL token introduces price volatility, a risk that could be mitigated in future iterations through the integration of stablecoins. To translate these cost savings into a standard business metric, the Return on Investment (ROI) for a healthcare provider adopting the system can be calculated using the framework in Equation 11.

(11) $$\mathrm{ROI}=\frac{\left(\mathrm{Annual}\;\mathrm{Savings}-\mathrm{Implementation}\;\mathrm{Cost}\right)}{\mathrm{Implementation}\;\mathrm{Cost}}\times 100\text{\%}$$

**Table 14:** Comparison of estimated EHR interaction costs across different systems and regions.

Region/System	Avg. cost per patient visit (INR)	Source/Basis
**PolyMed (India)**	₹**4.44**	On-chain gas cost analysis
US EHR Systems	₹50	Industry estimates
EU EHR Systems	₹250	Industry estimates
UK NHS Systems	₹150	Public data estimates
Singapore Systems	₹180	Industry estimates

## 7 Discussion

The empirical results from the performance, usability, and economic analyses demonstrate that PolyMed is a technically viable and highly efficient platform for decentralized EHR management. This section interprets these findings, discusses their broader implications for the healthcare paradigm, contextualizes the architectural choices made in the study, and frankly addresses the limitations of the current study and outlines key open research challenges.

### 7.1 Interpretation of key findings

The performance benchmarks presented in Section 4 are not merely technical metrics; they represent critical enablers for real-world clinical adoption. The sub-4-second transaction latency is particularly significant, as it falls within the acceptable threshold for interactive clinical workflows, where physicians need near-instant access to patient records during consultations or emergencies. Furthermore, the demonstrated throughput of handling over 800 concurrent users confirms the system's suitability for a medium-to-large hospital environment. When combined with the over 90% reduction in operational costs compared to traditional EHR systems, PolyMed presents a compelling economic case, directly addressing a major barrier to the digitization of healthcare in developing economies.

In parallel, the AI model's performance, validated through a rigorous 10-fold cross-validation, underscores the platform's clinical potential. The model's strong and stable discriminative power, evidenced by a Mean AUC of **0.8543** ± **0.0099**, confirms its ability to reliably distinguish between emergency and non-emergency patient states. Crucially, the model was calibrated for a high-sensitivity clinical use case, achieving a Mean Recall of **70.04%**. This demonstrates that the architecture can transition healthcare from a reactive to a proactive model by successfully identifying the majority of adverse events, a key step towards improving patient outcomes.

### 7.2 The movement towards a patient-centric economy

Beyond technical efficiency, PolyMed's core contribution is its architectural shift towards a patient-centric healthcare economy. Unlike most prior systems that focused primarily on the technical problem of data storage, PolyMed integrates governance and economic layers that empower patients as first-class citizens of the digital health ecosystem. The integration of a DAO for governance is a direct response to the ethical questions surrounding data sovereignty. While challenges such as the “DAO Literacy Gap” identified in the usability study exist, they are addressed by design through a concrete onboarding framework, providing a transparent and democratic path for patients to have a verifiable say in the policies that govern their own data.

Furthermore, the DeFi module addresses a fundamental socio-economic barrier to healthcare: access to funds for treatment. By automating microloans through transparent smart contracts, the system provides a tangible financial utility that is inextricably linked to the patient's health journey. This fusion of clinical, governance, and financial services within a single, trustless ecosystem represents a significant advancement. It reframes the EHR from a passive data repository into an active platform for patient empowerment, a concept largely unexplored in the systems detailed in Table 1. Crucially, this model also introduces important ethical considerations, such as mitigating predatory lending risks and defining fair policies for loan defaults, particularly for vulnerable patients. The DAO governance framework is designed to directly address these challenges by enabling the community to transparently set and enforce ethical, patient-centric lending parameters.

### 7.3 Limitations and open research challenges

Despite promising results, this study has several limitations that must be acknowledged and that pave the way for future research.

• **Limited Generalizability of AI Model:** The AI model, while achieving a robust and stable performance across a 10-fold cross-validation, was trained and validated on a single public dataset (PhysioNet/CinC 2012). Its performance may not generalize perfectly to different patient demographics or clinical settings. Future work is essential to validate the model on diverse, multi-institutional datasets, ideally using privacy-preserving techniques like Federated Learning.
• **Small-Scale and Skewed Usability Study:** The usability study provided valuable initial feedback but was limited to 20 participants who were primarily students or healthcare professionals with a degree of technical familiarity. A larger-scale study including a diverse cohort of real patients with varying ages, technical literacy, and demographic backgrounds is required to fully assess the platform's user experience.
• **Lack of a Clinical Pilot Study:** While the on-chain components were tested on the Polygon Mainnet, the entire system has not yet been deployed in a live clinical environment. A pilot study within a hospital or clinic is the critical next step to test the system's resilience, utility, and integration capabilities under real-world operational pressures.
• **Hardware and Connectivity Robustness:** The IoT data pipeline was validated using a prototype in a controlled environment. While a mitigation framework for connectivity and power issues was designed into the edge layer, a production-grade deployment would require extensive field testing to validate its real-world effectiveness against challenges such as intermittent network coverage and device power constraints.
• **Private Key Management:** Like all decentralized applications, PolyMed relies on users to securely manage their own cryptographic private keys. This remains a significant usability and security hurdle for non-technical users. Future work must explore the integration of more user-friendly key management solutions, such as smart contract wallets with social recovery mechanisms.
• **Legacy System Interoperability:** To achieve widespread adoption, seamless integration with existing Hospital Information Systems is crucial (22). A concrete roadmap is proposed to develop and validate a fully HL7 FHIR-compliant API layer. The plan involves a three-phase pilot program: **Phase 1 (Q2 2026):** Develop and test a read-only FHIR API in partnership with a mid-sized clinical partner, focusing on patient demographic and lab data. **Phase 2 (Q4 2026):** Expand the API to support write-back capabilities for prescriptions and clinical notes in a limited trial. **Phase 3 (2027):** Full integration trial with the partner's primary information system.
• **Global Identity Interoperability:** The current prototype's reliance on Anon-Aadhaar for ZKP-based identity verification, while effective for the Indian context, presents a clear limitation for global applicability. To evolve into a universally accessible platform, the modular “AuthSC” must be extended to support a wider range of verifiable identity standards. Future work will focus on integrating ZKP frameworks compatible with established national e-ID systems, such as the EU's eIDAS regulation, and embracing emerging W3C standards like Decentralized Identifiers (DIDs) and Verifiable Credentials (VCs). This would allow users worldwide to anchor their on-chain identity to their respective trusted national or decentralized credentials.

## 8 Conclusion and future work

This paper introduced PolyMed, a novel, decentralized architecture for patient-centric EHR management that addresses critical vulnerabilities in traditional centralized systems. By synergistically integrating the Polygon blockchain, validated AI, ZKP-based self-sovereign identity, and DAO-led governance, PolyMed establishes a secure, transparent, and efficient ecosystem for health data. The empirical evaluation confirms the platform's practical viability, demonstrating sub-4-second transaction latencies, significant cost reductions, and a clinically-calibrated AI-driven emergency detection model. The model's strong discriminative power (Mean AUC **0.8543**) was confirmed through a rigorous 10-fold cross-validation, demonstrating its potential to improve patient safety. The platform's privacy-by-design architecture is aligned with global data protection mandates, ensuring patient data sovereignty. PolyMed's holistic approach, combining clinical utility with patient empowerment, represents a significant step towards a more equitable and proactive digital health paradigm.

### 8.1 Future work

Building on the robust foundation established in this work, future research directions will focus on scaling, feature enhancement, and ecosystem expansion.

A key priority is to conduct a full-scale **clinical pilot study** in a partner hospital to validate the system in a real-world setting. Concurrently, the **HL7 FHIR-compliant interoperability bridge**, outlined in the limitations, will be developed following a phased roadmap to ensure seamless data exchange with legacy hospital systems. To prepare for large-scale adoption, an exploration of advanced **Layer-2 scaling solutions**, such as ZK-Rollups, could further enhance throughput and reduce costs.

Further research will also enrich the user experience and clinical utility. The use of SBTs will be expanded to create **portable medical credentials**, allowing records like immunization histories to function as secure, verifiable digital artifacts. Advanced **AI Clinical Assistants** could be deployed, leveraging transformer-based models for tasks like symptom triage and diagnostic support, while real-time **Clinical Visualization Dashboards** will provide clinicians with actionable insights. Critically, this phase will address the most significant usability and security hurdle for non-technical patients: user-managed private keys. The reliance on wallets like MetaMask creates a single point of failure leading to irreversible data loss. This will be mitigated by integrating advanced solutions such as **smart contract wallets** that enable user-friendly **social recovery** via designated guardians, and by exploring **Multi-Party Computation (MPC)**-based key management to eliminate the single point of failure entirely.

A long-term vision is to grow PolyMed into a self-sustaining digital health ecosystem. This includes expanding the DeFi module into a comprehensive **Decentralized Health Finance** platform, supporting community-funded microinsurance and peer-to-peer lending. A dedicated **ZK Analytics Engine** that utilizes optimized SNARK queries could be developed to allow researchers to perform complex, privacy-preserving analyses on aggregated health data. This will be complemented by the use of **Federated Learning** to train the AI models on diverse datasets without centralizing sensitive information. Finally, **Governance Expansion** will be pursued, enhancing the DAO's capabilities with formal mechanisms for on-chain policy audits and dispute resolution, ensuring the long-term stewardship of the platform.

With the digital healthcare landscape rapidly evolving toward decentralized architectures, PolyMed contributes a robust and ethically-sound framework for the next generation of globally interoperable eHealth systems.

## Data Availability

The raw data supporting the conclusions of this article will be made available by the authors, without undue reservation.
